# Ectopic B lymphocyte follicles exacerbate ischemic brain damage via MIF-CD74/CXCR4 and interferon signaling

**DOI:** 10.1172/JCI196905

**Published:** 2026-03-02

**Authors:** Sheng Yang, Hang Zhang, Lu-Lu Xu, Luo-Qi Zhou, Yun-Hui Chu, Lian Chen, Xiao-Wei Pang, Lu-Yang Zhang, Li-Fang Zhu, Ming-Hao Dong, Ke Shang, Jun Xiao, Long-Jun Wu, Wei Wang, Dai-Shi Tian, Chuan Qin

**Affiliations:** 1 Department of Neurology, Tongji Hospital, Tongji Medical College and State Key Laboratory for Diagnosis and Treatment of Severe Zoonotic Infectious Diseases;; 2 Key Laboratory of Vascular Aging, Ministry of Education, Tongji Hospital of Tongji Medical College; and; 3 Hubei Key Laboratory of Neural Injury and Functional Reconstruction, Huazhong University of Science and Technology, Wuhan, Hubei, China.; 4 Center for Neuroimmunology and Glial Biology, Institute of Molecular Medicine, University of Texas Health Science Center at Houston, Houston, Texas, USA.

**Keywords:** Immunology, Neuroscience, B cells

## Abstract

Neuroinflammation, encompassing both innate and adaptive immune responses, plays a crucial role in ischemic stroke. Although B lymphocytes are central to adaptive immunity, their contributions to ischemic stroke remain poorly understood. Here, we demonstrated that B lymphocytes accumulate in ischemic lesions, forming germinal center–like structures at the later stage after stroke, which mainly depended on in situ proliferation. This accumulation correlated with worsened neuroinflammation and ischemic injury, whereas B cell depletion reduced chronic brain damage during stroke. Mechanistically, microglia recruited B cells into ischemic lesions through MIF-CD74/CXCR4 signaling during the early phase of stroke, while IFN-related pathways in B cells further drove neuroinflammation and brain injury. Targeting these pathways markedly alleviated cerebral ischemia and inflammation. Our findings shed light on the role of B lymphocytes in stroke pathology and suggest promising new avenues for therapeutic intervention.

## Abstract 2

MIF-CD74/CXCR4 and interferon signaling pathway mediate both the recruitment of B lymphocytes by microglia and detrimental impacts of B lymphocytes on ischemic stroke

## Graphical abstract

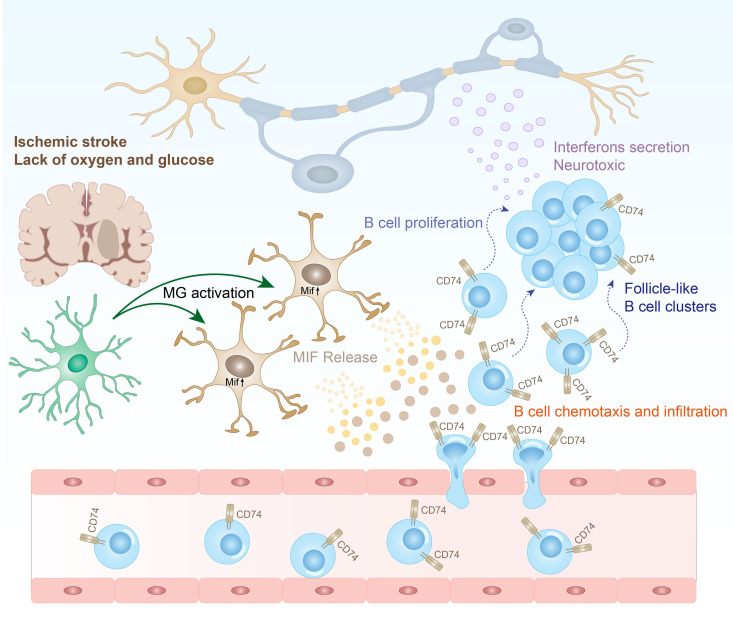

## Introduction

Ischemic stroke was the second leading cause of death (5.9 million deaths) and disability (102 million disability-adjusted life years lost) worldwide in 2010 (1, 2). Neuroinflammation plays a critical role in the pathogenesis of ischemic stroke (3). The immune response following ischemic stroke comprises both innate immunity, in which microglia orchestrate, and adaptive components, in which lymphocytes play crucial roles (3, 4). Notably, the innate and adaptive immune responses are closely interconnected, influencing each other reciprocally (5, 6). Further elucidation of the molecular mechanisms is needed for underlying these immune interactions.

B lymphocytes play pivotal roles in mediating humoral immunity and serve as critical links between the adaptive and innate immune systems (7). The roles of B lymphocytes have been documented in several CNS diseases, in which B lymphocytes exhibited abnormal immunological profiles and excessively produce proinflammatory cytokines and neurotoxic secretory factors (8, 9). However, the study of B lymphocytes in the context of ischemic stroke remains limited, and their phenotypic and functional alterations in ischemic lesions, as well as the impact on stroke pathology and underlying molecular mechanisms, require further investigation.

Microglia, the primary resident immune cells in the CNS, are crucial to innate immunity (10). Following ischemic stroke, microglia rapidly migrate to ischemic lesions, undergoing marked morphological and functional changes (11). They also play a role in attracting and recruiting other immune cells to these lesions through antigen presentation and the secretion of various cytokines and chemokines, as well as through mutual interactions with multiple ligand-receptors (12). The interactions between microglia and other immune cells may bridge the innate and adaptive immune responses. Nonetheless, the mutual interactions between microglia and B lymphocytes remain poorly understood.

This study employs both experimental ischemic stroke models and human patient samples to investigate the spatial and temporal changes of B lymphocytes and their effects on ischemic injury. In this study, we identify the MIF-CD74/CXCR4 signaling pathway as a critical mediator driving microglial recruitment of B lymphocytes to ischemic lesions. Additionally, B lymphocytes exacerbate neuroinflammation induced by cerebral ischemia by activating IFN-related signaling pathways, with blockade of these pathways resulting in reduced ischemic injury. Both the MIF-CD74/CXCR4 and IFN-related signaling pathways mediate interactions between microglia and B lymphocytes, presenting potential therapeutic targets for ischemic stroke.

## Results

### B lymphocytes form follicle-like structures in the later stage of ischemic stroke.

**Figure 1:**
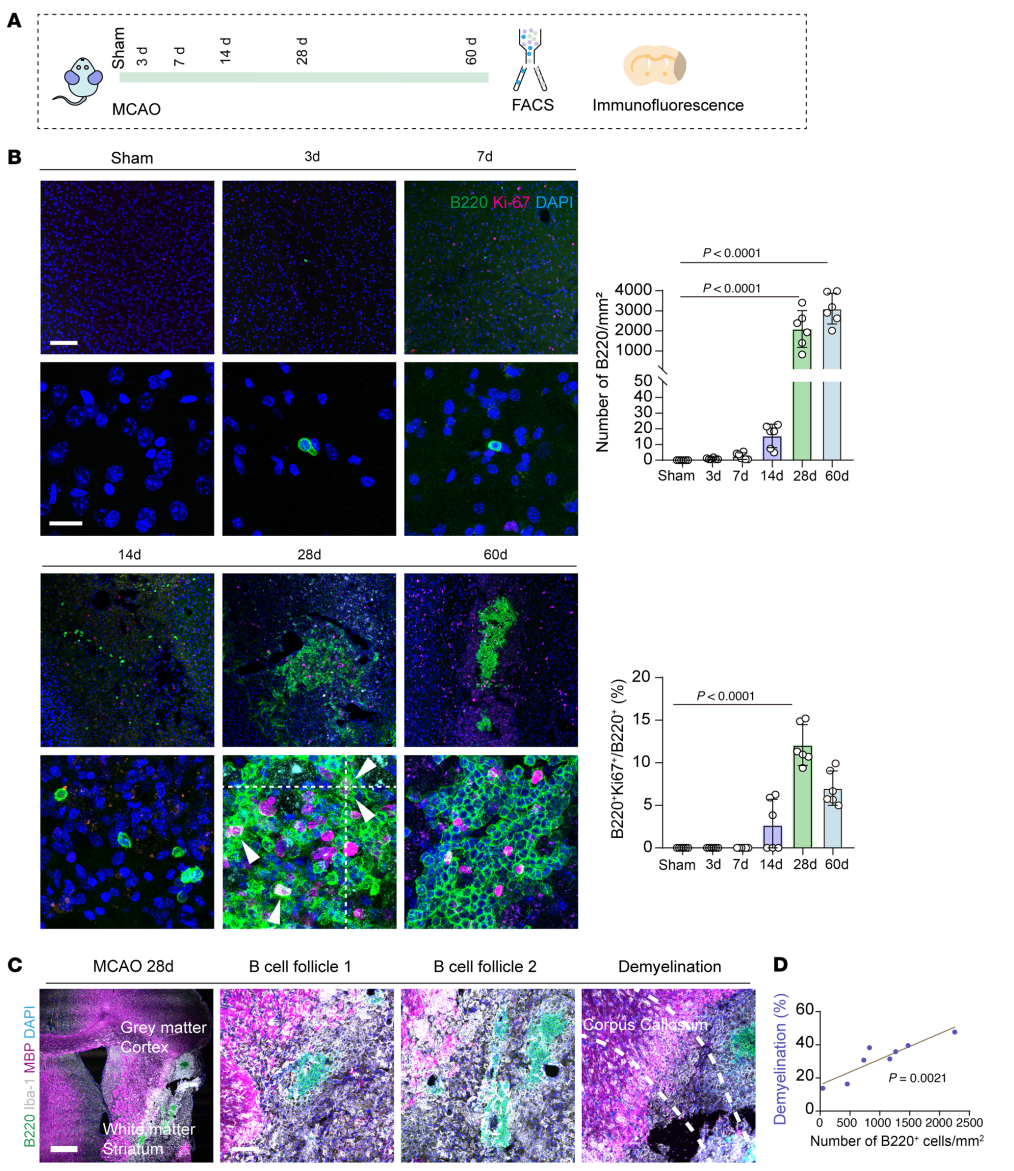
B lymphocytes massively accumulate into the ischemic lesions. (**A**) Schematic plot illustrating the experimental strategy of depicting temporal trend of B lymphocyte infiltration after ischemic stroke. (**B**) Illustration of B lymphocyte infiltration after ischemic stroke at different time points by costaining immunofluorescence of B220 and Ki67. *n* = 6 biological replicates per group. Scale bar: 100 μm for the upper panel and 20 μm for the lower panel. 1-way ANOVA followed by Bonferroni’s post hoc test. (**C** and **D**) Position of B lymphocyte clusters in ischemic lesions and the correlation between B lymphocyte infiltration and extent of white matter damage in ischemic stroke. *n* = 8 biological replicates. Scale bar: 500 μm (left) and 100 μm (right), respectively.

We performed immunofluorescence on mouse brain slices at various time points following middle cerebral artery occlusion (MCAO) (Figure 1A). Altogether 36 mice were analyzed in this analysis. At 3–7 days after stroke, a small number of B220^+^ B lymphocytes infiltrated the ischemic lesions, while at day 14, these cells remained sporadically distributed (Figure 1B). By day 28, the number of B220^+^ B lymphocytes had significantly increased compared with earlier time points (3, 7, and 14 days) (Figure 1B). Additionally, in the cerebral ischemic lesions, only a very small fraction of B lymphocytes was colocalized with CD31^+^ microvessels, which are almost sporadically distributed (Supplemental Figure 1A; supplemental material available online with this article; https://doi.org/10.1172/JCI196905DS1).

**Figure 2:**
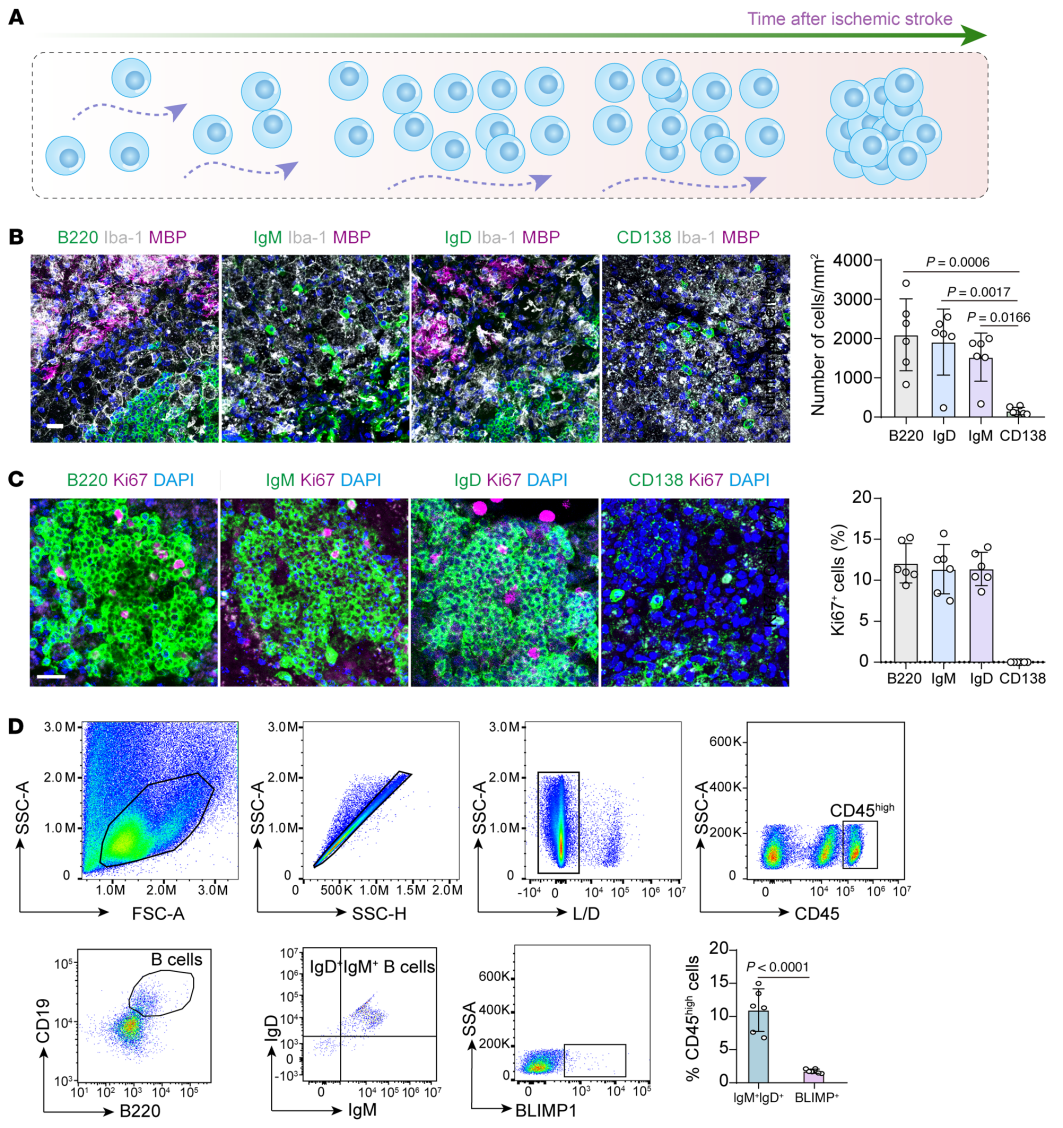
Characteristics of the massively accumulating B lymphocytes in the ischemic lesions. (**A**) Graphic illustrator showing the infiltration and accumulation processes of B lymphocytes in ischemic stroke lesions. (**B**) Immunofluorescence staining of B220, IgM, IgD, and CD138 to illustrate the proportion of B lymphocytes at different stages in ischemic stroke lesions. *n* = 6 biological replicates per group. Scale bar: 20 μm. 1-way ANOVA followed by Bonferroni’s post hoc test. (**C**) Immunofluorescence costaining of B220, IgM, IgD, and CD138 with Ki-67. *n* = 6 biological replicates per group. Scale bar: 20 μm. (**D**) Flow cytometry analysis revealing the proportions of IgM^+^IgD^+^ B lymphocytes and plasma cells (BLIMP1^+^ cells) in immune cells in ischemic lesions. *n* = 6 biological replicates per group. Unpaired 2-tailed *t* test.

A positive correlation was observed between the number of B lymphocytes in the lesions and the extent of ischemic demyelination, suggesting a potential detrimental role of these cells in ischemic stroke pathology (Figure 1, C and D). Further analysis of B lymphocyte characteristics in the ischemic hemispheres of stroke-affected mice revealed that, at 28 days after stroke, the majority of B lymphocytes were IgM^+^IgD^+^ mature cells. In contrast, plasma cells constituted much less proportion of the B lymphocyte population in the ischemic lesions, indicated by both immunofluorescence (CD138^+^) and flow cytometry analysis (BLIMP1^+^) (Figure 2, A–D).

**Figure 3:**
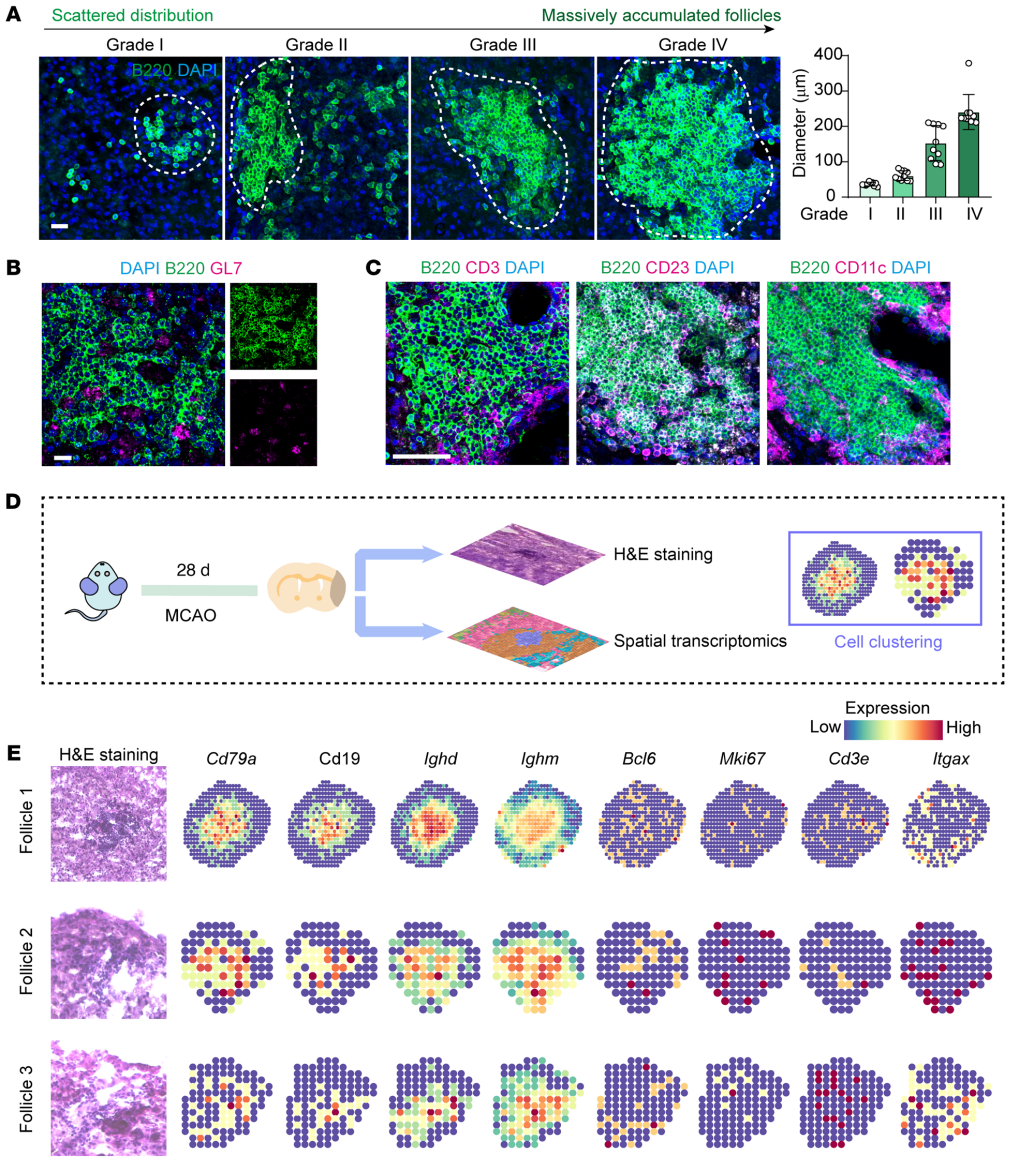
Spatial and cellular characteristics of massively accumulated B cells in the ischemic lesions. (**A**) Illustration of B lymphocyte clusters of different diameters in MCAO 28d group and the related diameter statistics. *n* = 10 clusters per group. Scale bar: 20 μm. The white dotted lines show the range of the follicle-like structures formed by B lymphocytes. (**B**) Costaining of B220 and GL-7 to illustrate the existence of follicle-like structures in B lymphocyte clusters. Scale bar: 20 μm. (**C**) Costaining of B220+CD3, B220+CD23, B220+CD11c immunofluorescence staining showing the spatial relationships between B cells, T lymphocytes, and dendritic cells. (**D**) Schematic plot showing the workflow of spatial transcriptomics analysis of ischemic lesions at 28d after MCAO. (**E**) Expression of markers of different B cell subclusters and representative markers for T cells (CD3e) and dendritic cells (Itgax) in the B cell massively accumulated regions.

We then further explored the spatial and transcriptomic characteristics of these massively accumulated B cells in the ischemic lesions. Interestingly, B lymphocytes in poststroke lesions exhibited varying distribution patterns, ranging from sporadic to extensive accumulation at day 28 (Figure 3A). Notably, at both day 28 and 60 after stroke, B lymphocytes in the ischemic lesions formed follicle-like structures within the cortex and striatum, accompanied by an elevated proportion of proliferative B lymphocytes (marked by Ki67^+^B220^+^) and the expression of GL7 (colocalized with a fraction of B lymphocytes), indicative of germinal center–like structure formation (Figure 2C and Figure 3B). Interestingly, the proportion of both GL-7^+^ and Ki67^+^ in IgM- and IgD-positive cells is comparable with that in the total B220^+^ B lymphocytes, suggesting that it’s the dominant IgM^+^IgD^+^ B cell that exhibits proliferative properties (Supplemental Figure 2 and Figure 2C). Moreover, by immunofluorescence staining, we’ve found that there’s also distribution of T cells (marked by CD3) and follicular dendritic cells (marked by CD23 and CD11c) that surround the massively accumulated B lymphocyte structures, indicating that these massively accumulated B cell structures resemble the follicle structures identified in tumor lesions as previously described (Figure 3, B and C) (13, 14). We then implemented spatial transcriptomic analysis on the brain hemispheres in mice 28 days after ischemic stroke. It can be revealed that these massively accumulated B cells express both markers for mature B cells (*Ighm* and *Ighd*, encoding IgM and IgD, respectively) and those for proliferation and germinal center formation (*Bcl6* and *Mki67*). Also, high expressions of markers for T cells (*Cd3e*) and dendritic cells (*Itgax*) are also exhibited surrounding the center of these B cell structures, which correspond to our previous results on immunofluorescence, showing that these massively accumulated B cell structures resembles the follicle-like structures reported previously, while there are also smaller ones referred to as ‘aggregates’ (13, 14) (Figure 3, D and E).

### The origin of massively accumulated B lymphocytes relies on in situ proliferation in ischemic lesions.

**Figure 4:**
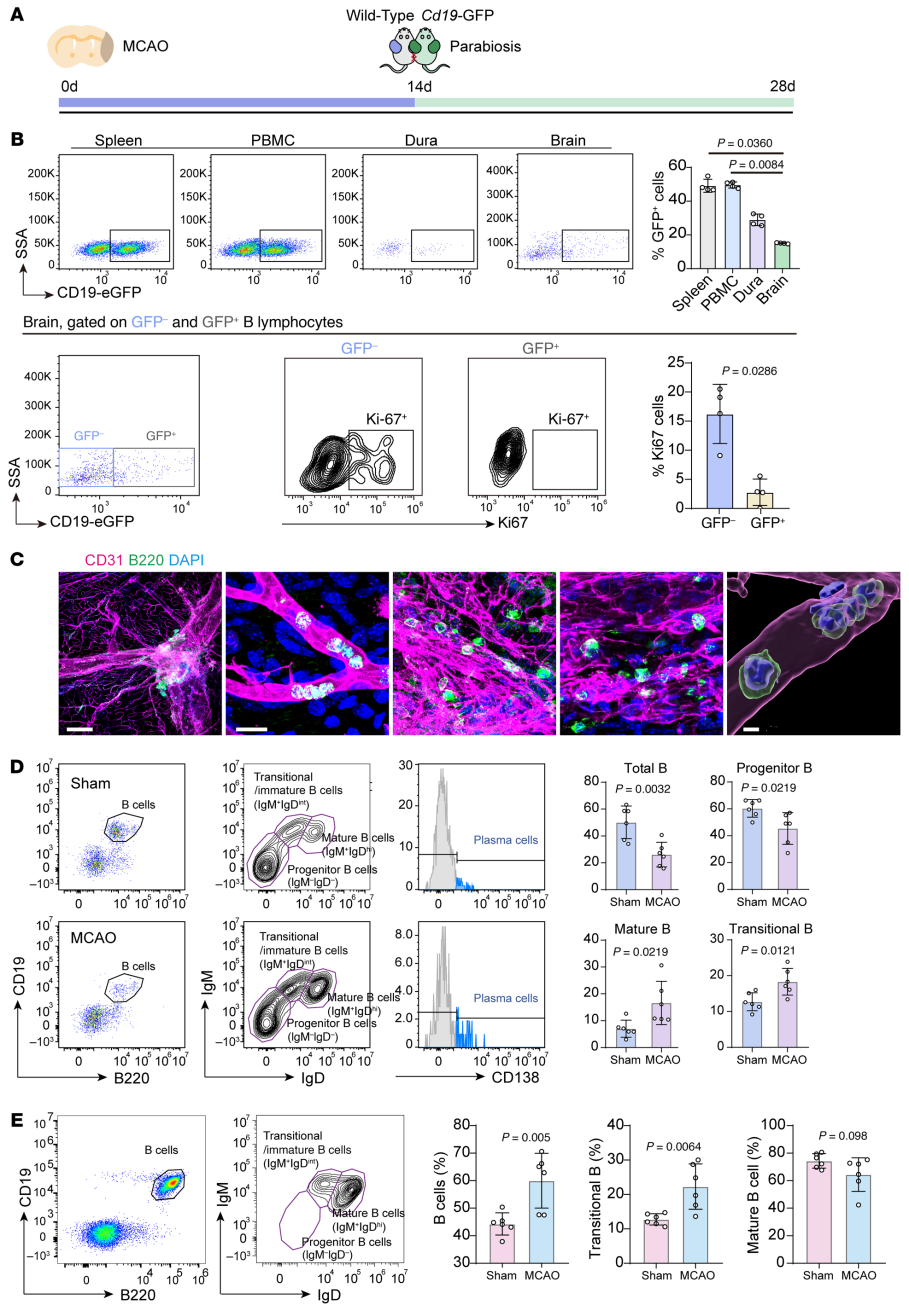
Origin of B lymphocytes in ischemic stroke and comparison between lesion-resident and periphery B lymphocytes. (**A**) Schematic plot illustrating 2 parabiosis experimental strategies involving WT mice and Cd19-DTR-eGFP transgenic mice, combined with MCAO surgery. (**B**) Proportion of GFP positive B lymphocytes in PBMCs, splenocytes, dura, and brains after ischemic stroke, and the proportions of Ki67^+^ cells in both GFP-positive and GFP-negative B cells specifically in the brain ischemic hemispheres in parabiosis strategy. *n* = 4 biological replicates per group. Kruskal-Wallis’ test followed by Dunn’s multiple comparison test for 4 group comparison and Mann-Whitney test for 2 group comparison, respectively. (**C**) B lymphocytes (green) in dura of mice, colocalized with CD31^+^ blood vessels (magenta). Scale bar: 500 μm (first panel; illustrating the whole dura), 20 μm (second panel), and 5 μm (final panel; 3D reconstruction figure by Imaris). (**D**) Analysis of B lymphocyte subclusters in dura from both sham-operated group and MCAO group. *n* = 6 biological replicates per group. Unpaired 2-tailed *t* test. (**E**) Analysis of B lymphocyte subclusters in PBMC from both sham-operated group and MCAO group, corresponding to Supplemental Figure 4. *N* = 6 biological replicates per group. Unpaired 2-tailed *t* test.

We have shown that there’s almost no infiltrating immune cells in the brain compared with PBMC and dura in sham-operated mice (Supplemental Figure 4). To determine the origin of the massive follicle-like B lymphocytes in ischemic lesions, we established a parabiosis model using *Cd19*-DTR-eGFP and WT mice, in which MCAO was first induced in both the WT mouse and *Cd19*-DTR-eGFP mice, and 2 weeks after MCAO, parabiosis was established between these 2 mice. We then analyzed the proportions of GFP^+^ B lymphocytes in the PBMCs, spleen, brain, and dura of the WT mice at 28 days after MCAO (Figure 4A).

In the parabiosis model, GFP^+^ B lymphocytes accounted for approximately 50% of the total B lymphocyte population in the PBMCs and splenocytes of WT mice. However, their proportion was significantly lower in the brain ischemic lesions and dura, indicating that the substantial accumulation of B lymphocytes in the ischemic lesions at later stages primarily derives from in situ proliferation rather than peripheral infiltration (Figure 4B and Supplemental Figure 15). Moreover, we’ve also compared the proportion of Ki67^+^ proliferating cells in both the GFP^+^ and GFP^–^ B lymphocytes. The results showed that, the proportion of Ki67^+^ proliferating cells is much higher in the GFP^–^ lymphocytes (indicating the autologous B lymphocytes that has already existed in the ischemic lesions) than in the GFP^+^ lymphocytes (the allogeneic ones that infiltrate into the ischemic lesions at later stages of ischemic stroke) (Figure 4B). Meanwhile, we have also performed Ki67^+^ staining based on parabiosis model in PBMCs. From the results, the proportions of Ki67^+^ B cells are relatively low in both the GFP^–^ and GFP^+^ B cells, and there’s no statistically significant difference between these 2 groups in the proportion of Ki67^+^ B lymphocytes. suggesting that the in situ proliferation in the ischemic brain lesions mainly exists in the autologous B lymphocytes, further strengthening our conclusions (Supplemental Figure 5).

Additionally, we further analyzed the characteristics of B lymphocytes in peripheral and dura tissues. Distinct differences were observed in the distribution of B lymphocyte populations across these tissues. For example, total B lymphocyte counts were lower in the dura of MCAO mice compared with sham-operated controls, whereas counts in PBMCs were elevated. The proportions of various B lymphocyte subclusters, including mature B lymphocytes, transitional B lymphocytes, pro-B lymphocytes, CD5^+^ B1a cells, and regulatory B lymphocytes (Bregs) also differed significantly between Sham and MCAO groups in different tissues (Figure 4, C–E; Supplemental Figure 3; and Supplemental Figure 6). It has to be noted that, in the dura tissues, there are an amount of immature B cells (characterized by IgM^–^IgD^–^). In line with the previous studies regarding this issue (15), the proportion of immature B cells is around 50%. Compared with the peripheral, the proportion of the immature B cells is relatively high, the functions of which remain to be further explored.

In summary, these findings suggest that the accumulation of B lymphocytes in the later stages of ischemic stroke relies predominantly on in situ proliferation rather than peripheral recruitment, exhibiting follicle-like structures with properties resembling germinal center.

### The accumulation of B cells exacerbates ischemic brain injury.

**Figure 5:**
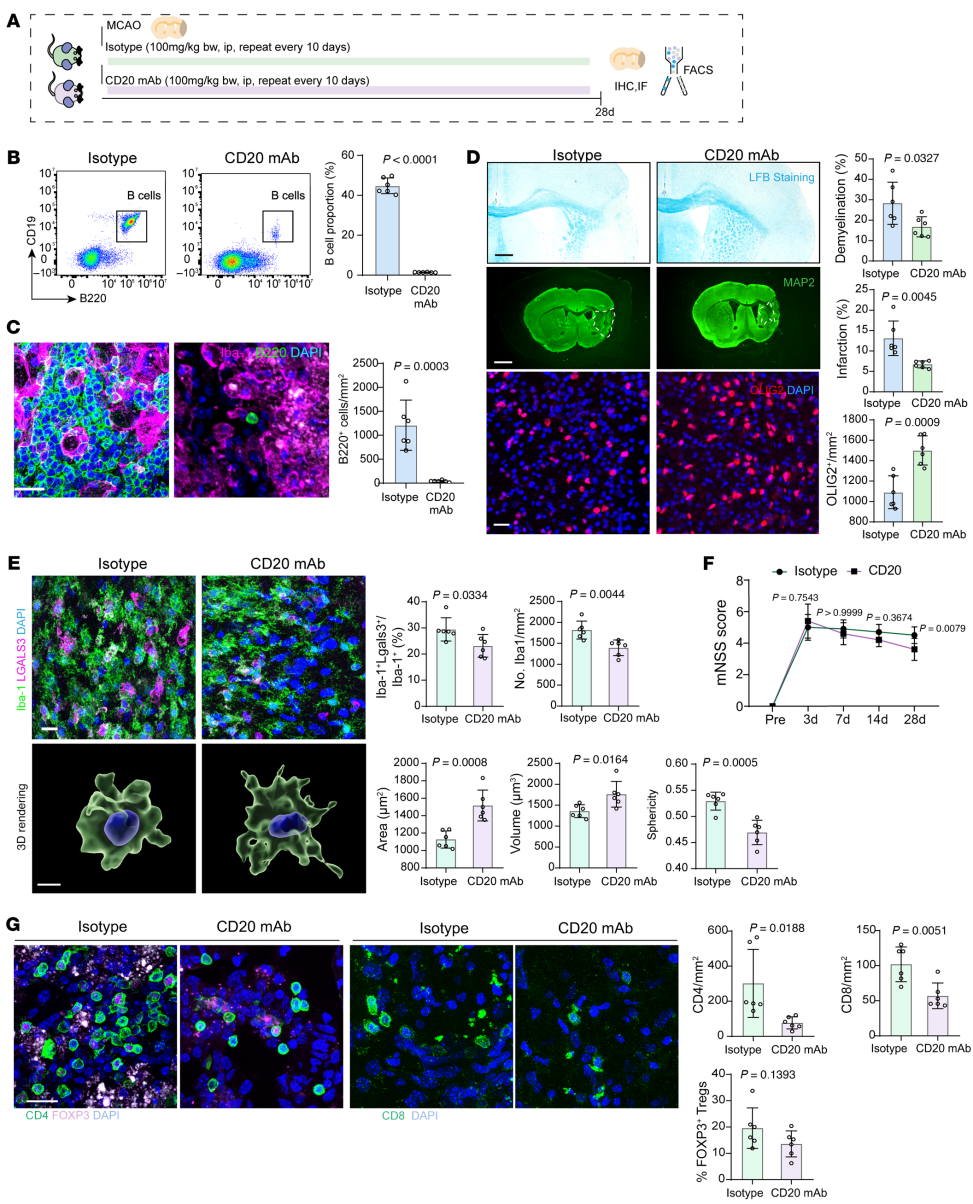
B lymphocytes depletion with anti-CD20 mAb reduces ischemic stroke injury and neuroinflammation. (**A**) Schematic diagram illustrating the strategy for depleting B lymphocytes using CD20 mAb in experimental ischemic stroke. (**B**) Flow cytometry validation of B lymphocyte depletion in the periphery after CD20 mAb treatment. *n* = 6 biological replicates per group. Scale bar: 20 μm. Unpaired 2-tailed *t* test. (**C**) Immunofluorescence validation of B lymphocyte depletion in cerebral ischemic lesions following CD20 mAb treatment. *n* = 6 biological replicates per group. Scale bar: 20 μm. Unpaired 2-tailed *t* test. (**D**) Effects of B lymphocyte depletion on cerebral ischemic injury, including comparisons of ischemic lesion size, demyelination areas, and oligodendrocyte proportions. *n* = 6 biological replicates per group. Scale bar: 500 µm for LFB and 1 mm for MAP2 staining. Unpaired 2-tailed *t* test. (**E**) Effects of B lymphocyte depletion on microglial morphology, status, and function at 28 days post-MCAO. *n* = 6 biological replicates per group. Scale bar: 20 μm. Unpaired 2-tailed *t* test. (**F**) Comparison of mNSS scores between groups at different time points post-MCAO. Analysis by 2-way ANOVA with Bonferroni post hoc test. *n* = 10 biological replicates per group. 2-way ANOVA followed by Bonferroni’s post hoc test. (**G**) Impact of B lymphocyte depletion on CD4^+^, CD8^+^ T lymphocyte and Treg cells infiltration at 28 days after MCAO. *n* = 6 biological replicates per group. Scale bar: 20 μm. Unpaired 2-tailed *t* test.

While we have identified the formation of follicle-like structures of B cells in lesions, their functional roles in ischemic stroke remain unclear. To explore this, we administered intraperitoneal injections of CD20 monoclonal antibody (mAb) to ablate B lymphocytes in mice following MCAO (Figure 5A). Flow cytometry and immunofluorescence analyses confirmed that the CD20 mAb effectively depleted B lymphocytes in both the peripheral blood and brain lesions (Figure 5, B and C).

The ablation of B lymphocytes led to improved neurological outcomes, reduced infarct size and demyelinated areas, and restored oligodendrocyte density in mice at 28 days after MCAO (Figure 5D). Microglia in the CD20 mAb-treated group showed a less activated state, as indicated by decreased sphericity and increased cellular volume and area (Figure 5E). Additionally, microglial phagocytic activity was reduced, evidenced by a lower proportion of LGALS3^+^ microglia (Figure 5E). These changes correlated with improved neurological function (mNSS score) after ischemic stroke (Figure 5F). Moreover, B cell depletion by CD20 mAb decreased the infiltration of both CD4^+^ and CD8^+^ T cells into the ischemic lesions, while the number of regulatory T cells remained unchanged (Figure 5G).

**Figure 6:**
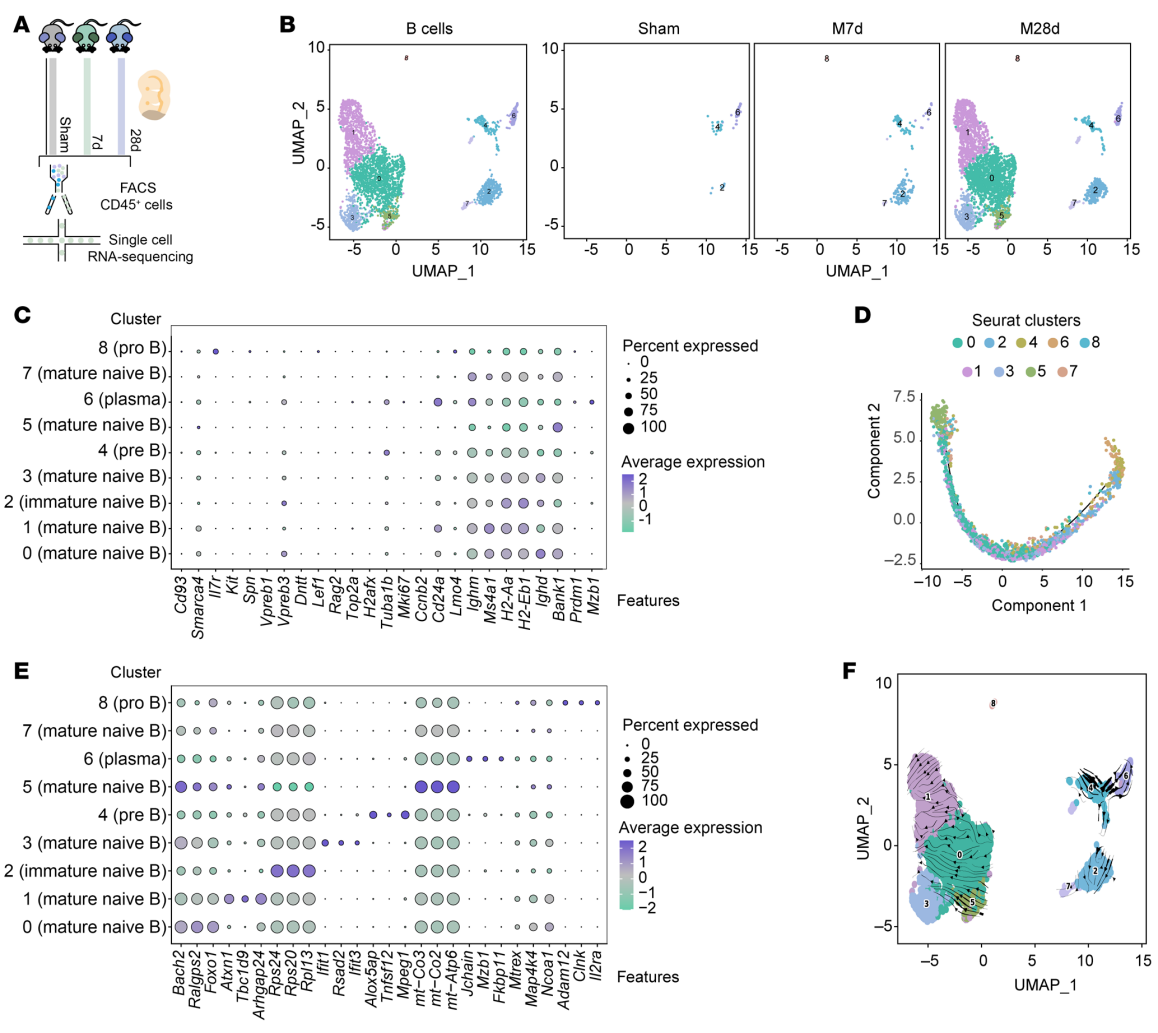
Transcriptional profiles of B lymphocytes in cerebral ischemic lesions after MCAO. (**A**) Schematic overview of the experimental strategy, including FACS-based cell sorting and subsequent scRNA-sequencing procedures. (**B**) Uniform Manifold Approximation and Projection (UMAP) plot of B lymphocytes in ischemic brain lesions. (**C**) Bubble plot depicting biomarkers of B lymphocyte differentiation for each B lymphocyte subcluster. (**D**) Pseudotime analysis of B lymphocytes in the brain at 28 days after MCAO. (**E**) Bubble plot depicting representative biomarkers for each B lymphocyte subcluster. (**F**) RNA-velocity analysis of B lymphocytes in the brain at 28 days after MCAO.

**Figure 7:**
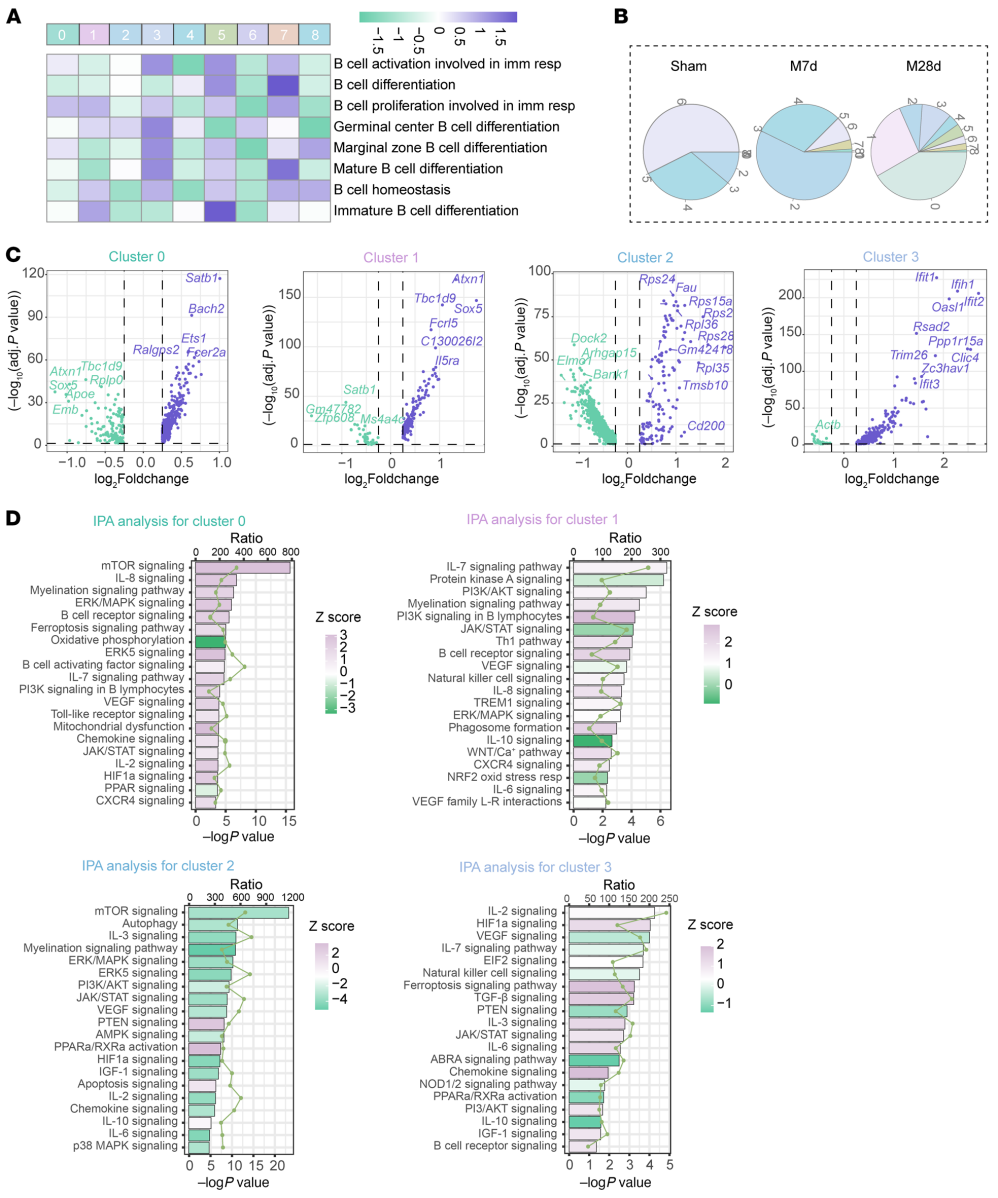
Biological functions of B lymphocytes based on transcriptional characteristics in cerebral ischemic lesions after MCAO. (**A**) Gene Set Variation Analysis (GSVA) showing enrichment scores for various biological processes across different B lymphocyte subclusters. (**B**) Proportions of B lymphocyte subclusters at different time points (Sham, MCAO 7d, and MCAO 28d). (**C**) Volcano plot displaying differentially expressed genes across B lymphocyte subclusters. (**D**) Ingenuity Pathway Analysis (IPA) of the transcriptional profiles for each B lymphocyte subcluster. *P* < 0.05 is considered statistically significant.

To further examine the nature of the accumulating B lymphocytes in ischemic lesions after MCAO, we performed scRNA-seq on CD45^+^ cells isolated from the ischemic hemispheres of 2 Sham, 3 MCAO 7-day, and 3 MCAO 28-day mice (Figure 6A and Supplemental Figures 7 and 13), in which altogether 58,958 cells were analyzed. Consistent with our immunofluorescence results, the number of B cells in the MCAO 28-day group was much higher than in the Sham and MCAO 7-day groups (Figure 6B). Subclustering analysis identified 9 B cell clusters, primarily consisting of mature B cells (Figure 6, B and C, and Supplemental Table 1). Plasma cells (Cluster 6, characterized by high expression of *Mzb1* and *Prdm1*) and progenitor B lymphocytes represented only a small fraction (Figure 6C and Figure 7B), corresponding with the results based on immunofluorescence and flow cytometry analysis (Figure 2). On the other hand, to show the validity of the clustering approach, the expression profiles of the representative biomarkers for different B lymphocyte subclusters were also illustrated in Figure 6E. Pseudotime analysis confirmed the differentiation trajectory, in which progenitor B lymphocytes was distributed at the origin of the trajectory and plasma cells was at the terminal part (Figure 6D). RNA velocity analysis also revealed that subcluster 4 and 8 are located at the most original position of the differentiation trajectory, on the one hand transforming toward cluster 6, and, on the other hand, toward clusters 2–7, and eventually to clusters 0, 1, 3 and 5, further confirming the results of B lymphocyte differentiation (Figure 6F). Notably, clusters 0, 1, 3, and 5 were predominant in the MCAO 28-day group, while Cluster 2 accounted for around 60% of B cells in the MCAO 7-day group, suggesting that Cluster 2 B cells may be the initial infiltrators into ischemic lesions (Figure 6B). Clusters 0, 1, 3, and 5 likely contribute to the formation of follicle-like structures observed in later stages of stroke.

Differential gene expression analysis was performed to distinguish differentially expressed biomarkers of different B lymphocyte subclusters based on all the B lymphocytes (Figure 7C). Functional analysis using Ingenuity Pathway Analysis (IPA) revealed distinct roles for different subclusters. Clusters 0, 1, and 3 exhibited transcriptional profiles associated with B cell activation, proliferation, and the secretion of proinflammatory cytokines and chemokines, indicating their potential proinflammatory roles. Cluster 6 exhibits high expression of markers such as *Prdm1*, *Mzb1,* and *Jchain*, and is termed the plasma cells. In contrast, subcluster 2 showed lower levels of activation but higher expression of ribosome-related molecules, as well as enhanced cell death–related pathways, such as PTEN and apoptosis signaling, along with interacting with other cells and inducing death processes, in which molecules such as *Cd74, H2-D1, H2-K1,* and *H2-Eb1* are involved (Figure 7, A, C, and D, and Supplemental Figure 8).

### MIF-CD74/CXCR4 signaling pathway mediates B lymphocyte recruitment to ischemic brain lesions.

**Figure 8:**
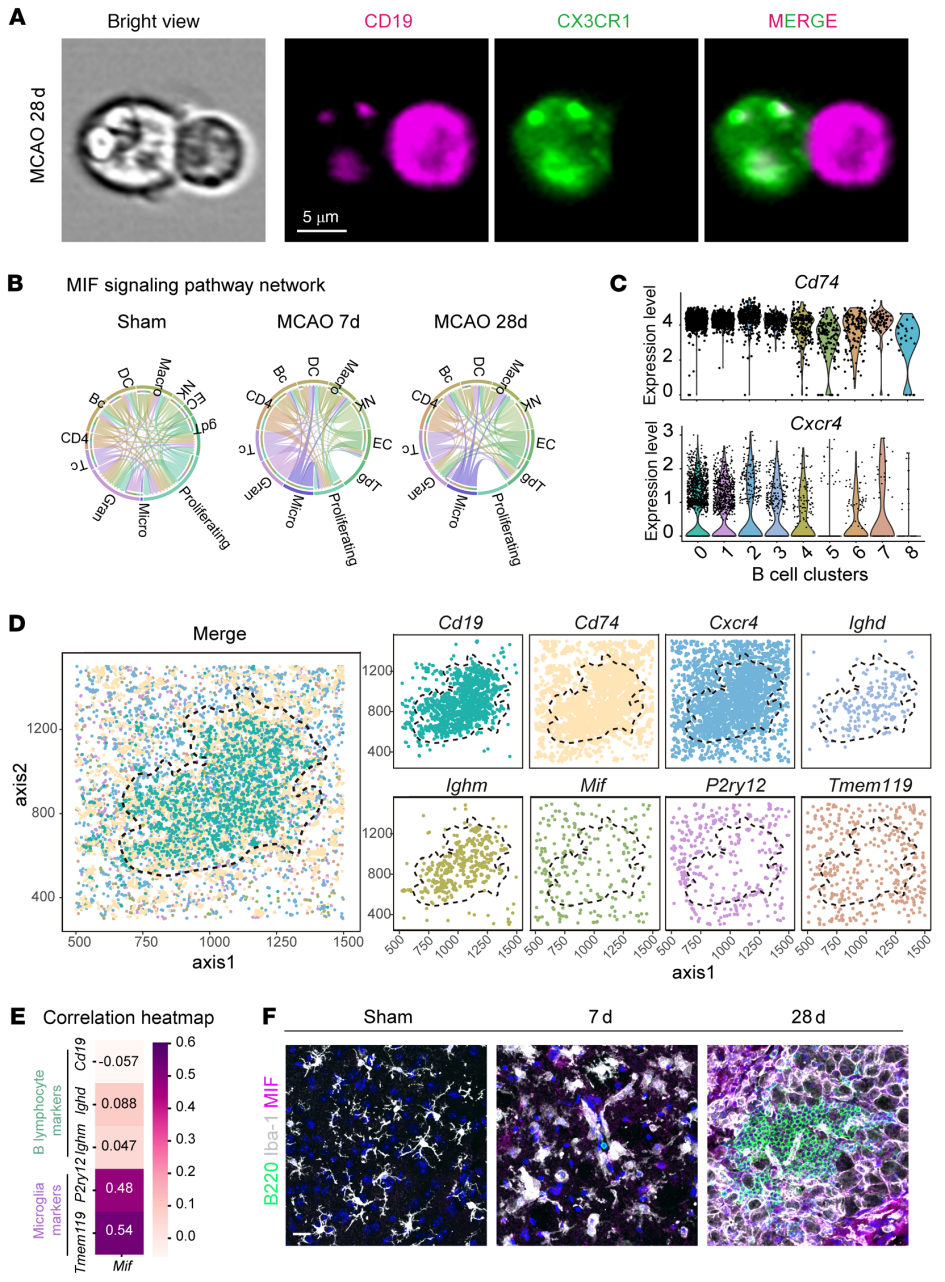
MIF-CD74/CXCR4 signaling pathway orchestrates interactions between microglia and B lymphocytes in ischemic stroke. (**A**) Imaging flow cytometry plots by Amnis multispectral imaging flow cytometry illustrating the spatial interaction between B lymphocytes and microglia in ischemic lesions. (**B**) CellChat analysis depicting microglia-B lymphocyte interactions via the MIF-CD74/CXCR4 signaling pathway at different time points (Sham, MCAO 7d, and MCAO 28d). (**C**) Expression levels of Cd74 and Cxcr4 across different B lymphocyte subclusters. (**D**) Multi-channel FISH analysis showing expression patterns of key markers involved in the MIF-CD74/CXCR4 signaling pathway in microglia and B lymphocytes. (**E**) Correlation analysis of the expression level of *Mif* with both B lymphocyte and microglia biomarkers. (**F**) MIF expression in microglia and its spatial proximity to B lymphocytes within ischemic lesions. Scale bar: 20 μm.

To investigate how B lymphocytes are recruited to ischemic lesions, we focused on microglia, the resident immune cells of the central nervous system (CNS). Imaging flow cytometry revealed direct interactions between microglia and B lymphocytes in ischemic lesions 28 days after MCAO (Figure 8A). CellChat analysis indicated that, in the MCAO 7-day group, microglia interact with B cells through the MIF-CD74/CXCR4 signaling pathway, which sustains in the MCAO 28-day group, suggesting a crucial role in mediating these interactions (Figure 8B and Supplemental Figure 10).

We further examined the expression levels of molecules within the MIF-CD74/CXCR4 pathway. Notably, both *Cd74* and *Cxcr4* exhibited the highest expression in B cell subcluster 2 (Figure 8C), which constituted over half of the B cells in the MCAO 7-day group (Figure 7B). This finding indicates that B cells expressing high levels of CD74 and CXCR4, the receptors for MIF, are recruited early during ischemic stroke. Multichannel FISH analysis corroborated these results, showing MIF levels correlating with microglial markers (*P2ry12*, *Tmem119*), which is also illustrated by correlation analysis (Figure 8, D and E). Additionally, microglia from both the MCAO 7-day and MCAO 28-day groups displayed upregulated expression of MIF, surrounding B lymphocytes in the ischemic lesions (Figure 8F). Moreover, based on a publicly available database (GSE122709), the expression levels of MIF were also significantly elevated in PBMCs of both acute and subacute ischemic stroke patients compared with healthy controls, suggesting the roles MIF-related signaling pathways play in cerebral ischemia (Supplemental Figure 11 and Supplemental Tables 6 and 7).

**Figure 9:**
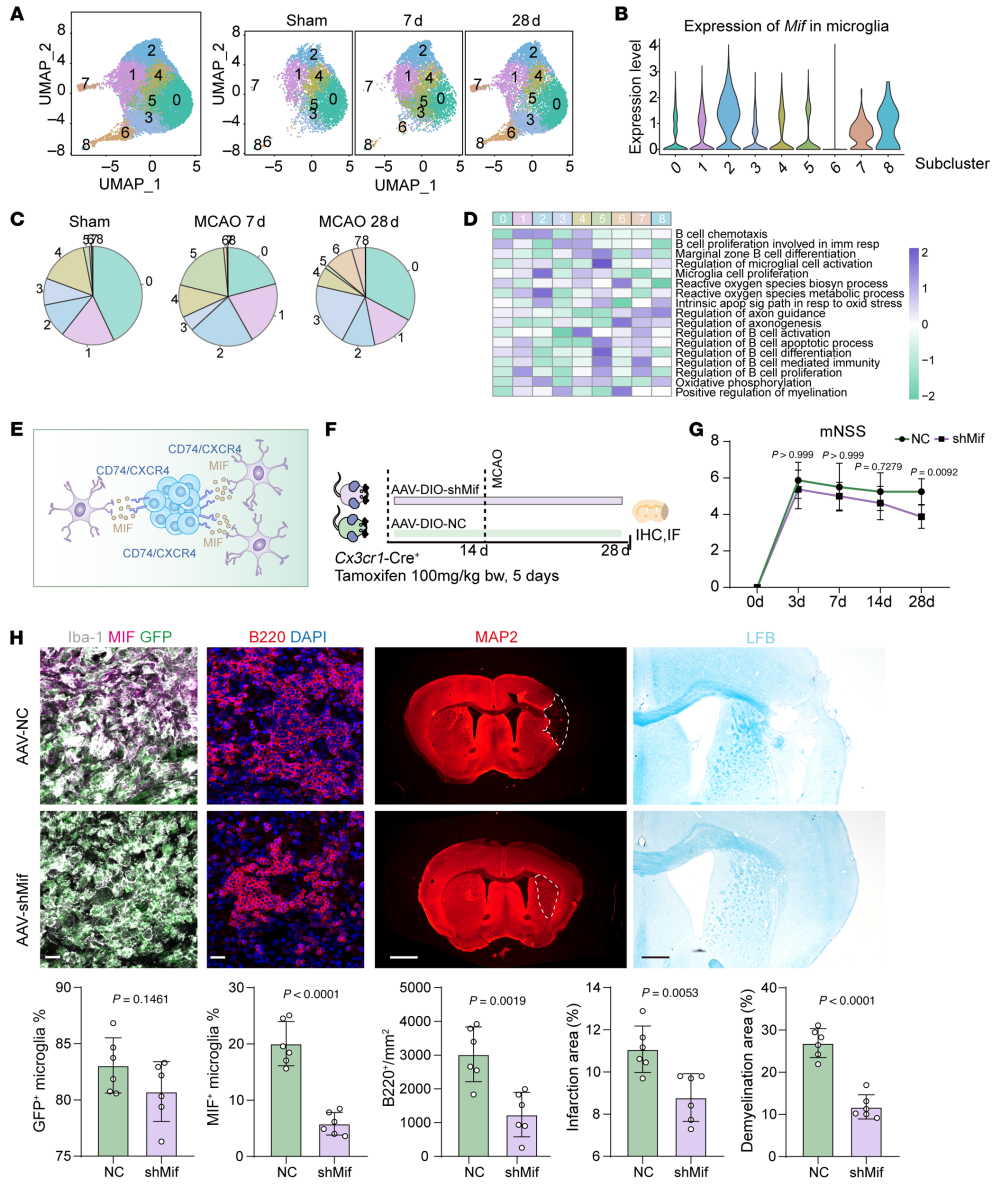
Effects of MIF-CD74/CXCR4 signaling pathway on ischemic stroke. (**A**) UMAP plot illustrating microglial subclusters at different time points (Sham, MCAO 7d, and MCAO 28d) after stroke. (**B**) *Mif* expression levels in various microglial subclusters. (**C**) Proportions of microglial subclusters across different time points (Sham, MCAO 7d, and MCAO 28d). (**D**) Gene Set Variation Analysis (GSVA) showing scores for biological processes and pathways in different microglia subclusters. (**E**) Schematic illustration of the microglia-B lymphocyte interactions mediated through the MIF-CD74/CXCR4 signaling pathway in ischemic lesions. (**F**) Schematic overview of the experimental strategy and study design for silencing microglial *Mif* to assess its effects on B lymphocyte infiltration, accumulation, and ischemic injury. (**G**) Impact of microglial *Mif* silencing on neurological deficits after cerebral ischemic stroke, *n* = 8 biological replicates per group, 2-way ANOVA followed by Bonferroni’s test. (**H**) Effects of microglial Mif silencing on ischemic brain damage and B lymphocyte infiltration. *n* = 6 biological replicates per group. Scale bar: 20 μm for immunofluorescence staining, 500 μm for LFB and 1 mm for MAP2 staining. Unpaired 2-tailed *t* test.

To further characterize microglial responses, we classified microglia into 9 subclusters using single-cell RNA-seq (Figure 9A and Supplemental Figure 9). MIF, as the ligand for CD74/CXCR4, was found to have the highest expression in microglial cluster 2 (Figure 9B and Supplemental Figure 10). This microglial subcluster exhibited the highest scores for B cell chemotaxis, proliferation, and oxidative phosphorylation, indicating its role in B lymphocyte recruitment (Figure 9, C–E). Additionally, this subcluster displayed molecular patterns resembling disease-associated microglia (DAM), along with upregulation of aerobic respiration processes, suggesting activated properties and potential detrimental effects (Supplemental Figure 21 and Supplemental Tables 5 and 8).

To elucidate the role of the MIF-CD74/CXCR4 pathway in ischemic stroke, we employed the DIO-AAV system and CreER transgenic mice, in which *Mif* is specifically knocked down in microglia to test the effects microglial *Mif* exerts on ischemic stroke outcomes (Figure 9F). Microglia-specific knockdown of *Mif* significantly alleviated ischemic injury, as evidenced by lower mNSS scores and reduced infarction areas (Figure 9, G and H). Additionally, there were no statistically significant differences in infarction areas and demyelination areas between these 2 groups at day 3, showing that silencing *Mif* specifically in microglia at the early time point had no significant impacts on ischemic injuries (Supplemental Figure 16). Notably, the number of B cells was also significantly reduced in the *Mif*-knockout group 28 days after MCAO (Figure 9H). In vitro coculture of microglia and B lymphocytes also revealed that, after *Mif* over-expression in microglia, the infiltration of B lymphocytes is significantly increased while silencing of *Cd74* in B lymphocytes significantly reversed these effects, suggesting the impacts of microglia-secreted MIF on the chemotaxis of B lymphocytes via CD74 signaling (Supplemental Figure 17). Moreover, we conducted scRNA-seq analysis in these 2 groups. From the results shown, firstly, in correspondence to the immunofluorescence results, the proportion of B lymphocytes is lower after *Mif* silencing in microglia, and the expression of *Cd74* in B lymphocytes is significantly decreased. Also, the expression levels of a variety of genes related to activation and biological activities including proliferation, differentiation in B lymphocytes — such as *Ighd*, *Cd86,* and *Bank1* — are also decreased after microglial *Mif* depletion, suggesting that silencing of microglia-B lymphocyte MIF-CD74 signaling pathway potentially dampened B lymphocyte chemotaxis, as well as proliferation and differentiation activities (Supplemental Figure 18).

Collectively, these findings showed that microglia possibly recruit B lymphocytes to ischemic lesions via the MIF-CD74/CXCR4 signaling pathway, suggesting a potential therapeutic target for ischemic stroke.

### The detrimental impacts of B lymphocytes are dependent on IFN-related signaling pathways.

**Figure 10:**
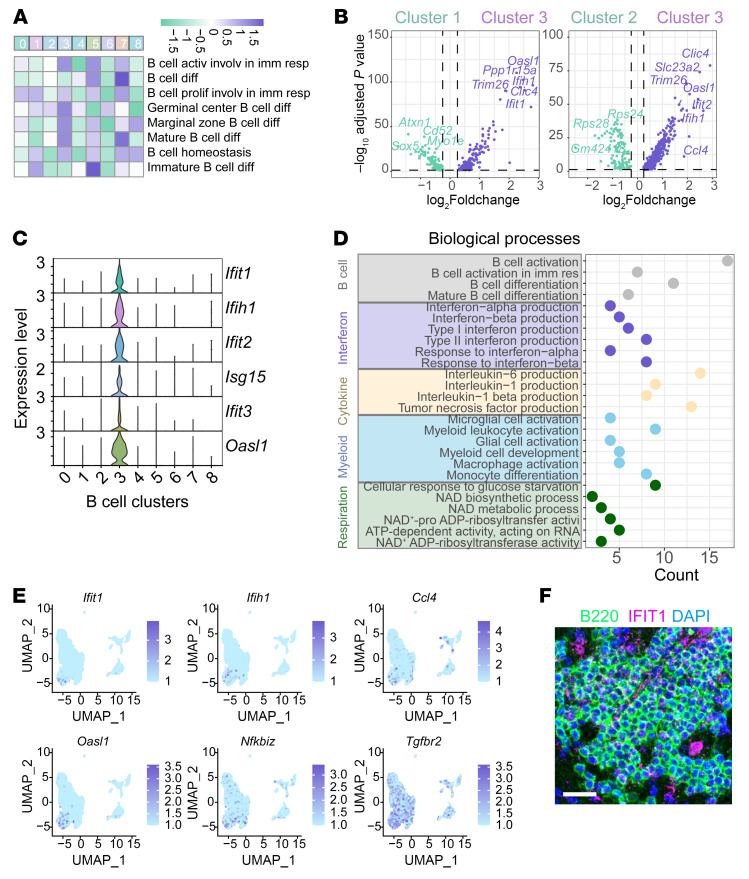
IFN-related signaling pathways and the relationships with B lymphocyte biological functions in ischemic stroke. (**A**) Gene Set Variation Analysis (GSVA) showing enrichment scores for various biological processes across different B lymphocyte subclusters, corresponding with Figure 7A. (**B**) Volcano plot illustrating the differentially expressed genes between microglial subcluster 3 and 1 and subcluster 3 and 2, respectively. (**C**) Expression levels of IFN signaling–related genes in B lymphocyte subclusters. (**D**) Biological processes enriched in B lymphocyte subcluster 3 with *P* < 0.05. ‘Count’ stands for the number of genes included in each item. (**E**) UMAP plots depicting the expression levels of IFN-related genes in B lymphocytes. (**F**) Immunofluorescence staining of IFIT1, a key marker of IFN-related signaling pathways in B lymphocytes within ischemic lesions. Scale bar: 20 μm.

To elucidate the mechanisms by which B lymphocytes contribute to cerebral ischemic injury, we utilized scRNA-seq and gene set variation analysis. B cell cluster 3 exhibited the highest scores for germinal center B cell differentiation and marginal zone B cell differentiation, highlighting its crucial role in forming follicle-like structures in postischemic lesions (Figure 10A and Supplemental Table 2). Also, the expression levels of germinal center formation–related genes, such as *Bcl6, Nfkbiz, Peli, Atm*, and *Ptprc*, are the highest in subcluster 3 (Supplemental Figure 19). Differentially expressed gene (DEG) analysis identified key biomarkers for subcluster 3, including *Ifit1, Ifih1, Ifit2,* and *Oasl1*, all associated with IFN activity (Figure 10, B and C). This led us to hypothesize that these abundant B cells, exhibiting germinal center–like properties, may exert detrimental effects on the CNS microenvironment through activation of IFN-related signaling pathways. Immunofluorescence confirmed the expression of IFN-related signals in part of the B lymphocytes in the follicle-like structures (Figure 10F). Further analysis of biological processes enriched in B lymphocyte subcluster 3 revealed high associations with IFN activities, B cell activation, proinflammatory cytokine release, and microglia/macrophage activation, along with respiratory chain activities (Figure 10, D and E, and Supplemental Table 3).

**Figure 11:**
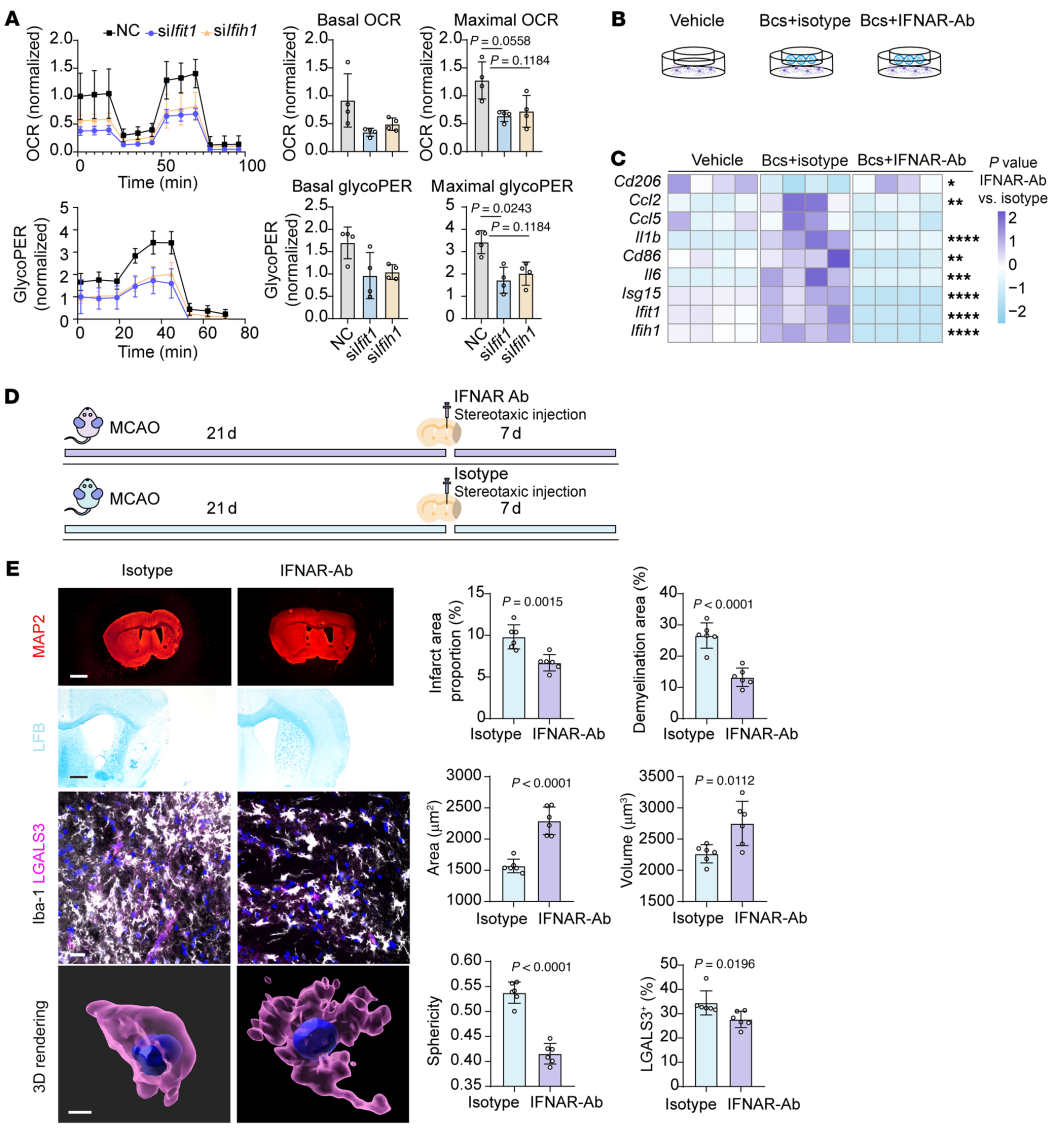
IFN-related signaling pathways drive the detrimental effects of B lymphocytes in ischemic stroke. (**A**) Seahorse analysis of B lymphocytes under different mRNA silencing conditions, showing results from glycolytic rate and mitostress assays. *n* = 4 biological replicates per group. Scale bar: 20 μm. Kruskal-Wallis test followed by Dunn’s multiple comparison test. (**B**) Schematic plot revealing the experiment design of the in vitro analysis regarding IFNAR blockade. (**C**) Effects of IFNAR blockade on microglia co-cultured with B lymphocytes, assessed by qRT-PCR. *n* = 4 biological replicates per group; *P < 0.05; **P < 0.01; ***P < 0.001; ****P < 0.0001. 1-way ANOVA followed by Bonferroni’s post hoc test. (**D**) Schematic plot revealing the experiment design of the in vivo analysis regarding IFNAR blockade. (**E**) Blocking IFN-related signaling pathways reduces ischemic brain injury and neuroinflammation. *n* = 6 biological replicates per group. Scale bar: 20 μm for immunofluorescence staining, 500 μm for LFB, and 1 mm for MAP2 staining. Unpaired 2-tailed *t* test.

Next, we investigated whether inhibiting IFN-related signaling could mitigate B cell–induced detrimental effects both in vivo and in vitro. We first primarily isolated high-purity B lymphocytes from mouse splenocytes (Supplemental Figure 12). Silencing IFN-related molecules, such as Ifit1 and Ifih1, led to reduced oxidative phosphorylation (measured by OCR) and glycolysis (measured by glycoPER) in B lymphocytes (Figure 11A). Coculture with B lymphocytes resulted in elevated proinflammatory profiles in primary microglia, the effects of which were significantly reduced by blocking IFNAR with a monoclonal antibody (Figure 11, B and C). We then intracerebroventricularly injected IFNAR monoclonal antibody in mice after MCAO (Figure 11D). Acute blockade of IFNAR alleviated cerebral ischemic injury (indicated by infarction area from MAP2 staining and demyelination area from LFB staining) and reduced neuroinflammation (assessed by microglia density, morphology, and phagocytosis activities) in ischemic lesions (Figure 11E). Meanwhile, the density of B lymphocytes in the aggregate structures showed the tendency of decrease after blockade of IFNAR, with no statistical significance found (Supplemental Figure 20).

**Figure 12:**
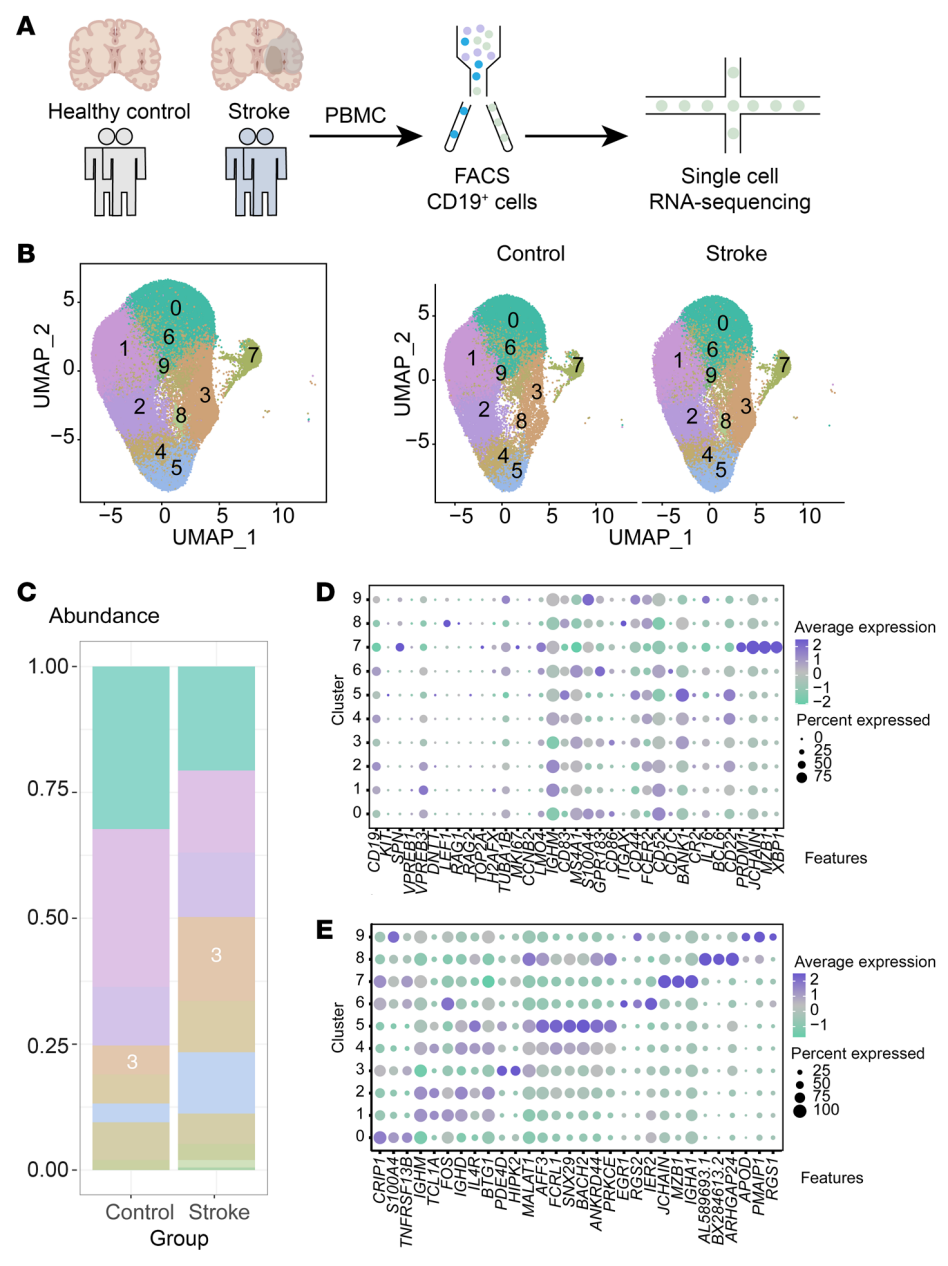
Validation of IFN-related signaling pathways in human PBMC samples of ischemic stroke. (**A**) Schematic plot illustrating the strategy for human B lymphocytes purification and single cell RNA Sequencing. (**B**) Uniform Manifold Approximation and Projection (UMAP) plot for B lymphocytes in human samples. (**C**) Proportions of different B lymphocyte subclusters in the Healthy Control and ischemic stroke groups. (**D**) Expression levels of biomarkers related with B cell differentiation for various B lymphocyte subclusters. (**E**) Representative biomarkers and expression levels for various B lymphocyte subclusters.

**Figure 13:**
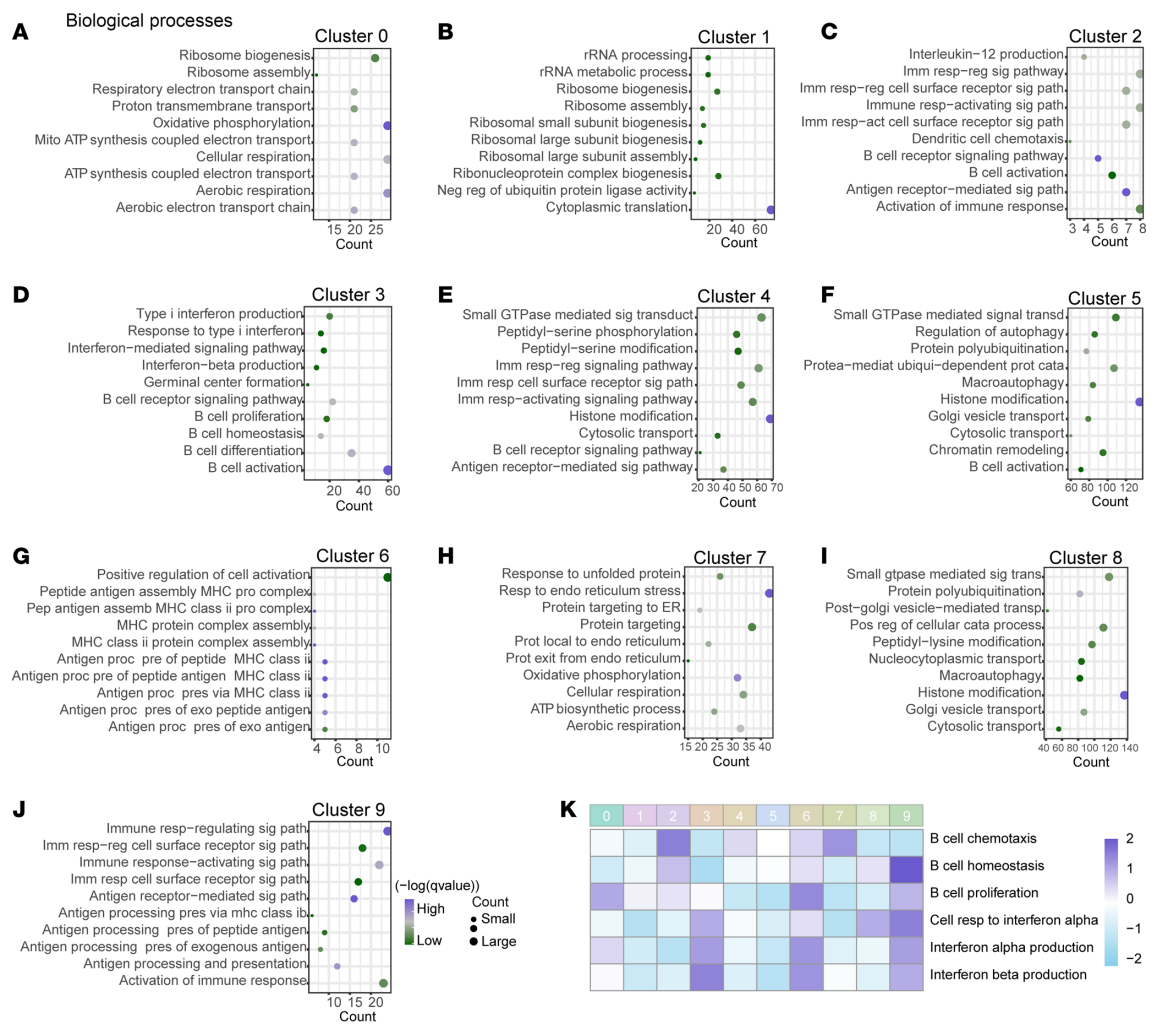
Validation of IFN-related signaling pathways in human PBMC samples of ischemic stroke. (**A**–**J**) Significant biological processes enriched in B lymphocyte subclusters 0–9. *P* < 0.05 and *q* < 0.05 were selected as the threshold for these biological processes. (**K**) GSVA showing scores for different biological processes and pathways associated with B lymphocyte activity and IFN-related signaling.

We also observed the change of IFN-related signaling pathways in ischemic stroke patients. In total, 72,627 B lymphocytes were sorted from PBMCs of both 4 healthy controls and 4 patients who had had ischemic strokes using FACS (Figure 12A and Supplemental Figure 14) and were subsequently utilized for further scRNA-seq analysis. scRNA-seq revealed that these B lymphocytes could be classified into several subclusters with distinct transcriptional profiles and differentiation status, illustrated by their representative biomarkers (Figure 12, B, D, and E). Next, we then studied the biological processes of each B lymphocyte subcluster in this dataset. Briefly, clusters 0 and 1 are enriched into ribosome-related pathways, as well as high energy demand. Cluster 2 is related with B cell activation, as well as immune-related signaling pathways. Cluster 3 is related with IFN-related activities, as well as B cell proliferation and germinal center formation. Both clusters 4 and 5 are associated with B cell activation and the related signaling pathways, as well as GTPase-mediated signal transduction and histone modification. Clusters 6 and 9 are related with antigen processing and presentation, while cluster 7, which is plasma cells, shows enhanced activities in protein production and transportation, along with ATP-related processes and cellular respiration. Likewise, cluster 8 is also related with biological processes such as GTPase mediated signal transduction, histone modification, and others (Figure 13, A–J).

Moreover, GSVA indicated that subcluster 3 exhibited high score for IFN-related signaling pathways, with a higher proportion found in ischemic stroke patients (Figure 12C and Figure 13K). Subcluster 3 was enriched for IFN production and B cell activation, as well as a potential role in germinal center formation, mirroring results from experimental ischemic stroke (Figure 13K and Supplemental Table 4).

Taken together, although there may be discrepancies between human and murine samples, our results show that the transcriptional signatures of human B lymphocyte subclusters could potentially be related to those in mouse B lymphocyte subclusters, which could be further explored in future studies.

## Discussion

In this study, we discovered that B lymphocytes accumulate in ischemic lesions, forming germinal center-like structures by 28 days after stroke. The formation of these follicle-like structures primarily relies on in situ proliferation. Microglia recruit B lymphocytes via the MIF-CD74/CXCR4 signaling pathway early in the ischemic process. Silencing microglial Mif expression alleviates B lymphocyte accumulation and activities, thereby protecting against cerebral ischemic injury. Notably, the B lymphocyte subcluster most associated with germinal center formation exhibits upregulated IFN-related transcriptional profiles. Inhibition of these IFN-related signals ameliorated ischemic injury and neuroinflammation both in vivo and in vitro, highlighting potential therapeutic targets against ischemic stroke.

B lymphocytes are crucial components of the adaptive immune system and have been implicated in various CNS diseases (16, 17). The roles of B lymphocytes are seemingly controversial, as results from one recent study suggest that anti-BCMA CAR-T therapy alleviated neuroinflammation in patients with MS (18). Conversely, one study showed that elimination of meningeal B cell aggregates with anti-CD19 chimeric antigen receptor–T cells abrogated disease outcome in opticospinal encephalomyelitis mice (19). The effects of B cell–related therapy in mice cannot be completely related to that in human studies, as one study revealed that the aforementioned exacerbation impacts of anti-CD19 CAR-T therapy were not observed in human patients with MS, suggesting its tolerable safety with promising disease-specific target-cell effects in patients with MS (20). Reportedly, higher number of (Un)switched memory B cells is associated with better outcome following carotid artery endarterectomy (21). Also, Kowarik et al. have found that CXCL13 serves as the major determinant to recruit B cells into the CNS compartment in different neuroinflammatory diseases (22), whereas another study has shown that CXCL12 and the related signaling pathways mediated B lymphocyte chemotaxis in the CNS (23), further suggesting the differences between mouse models and human patients.

In line with the previous study that showed the occurrence and effects of B lymphocytes in delayed stage of ischemic stroke (24, 25), our study identified B lymphocyte–formed follicle-like clusters in ischemic lesions at 28 days after stroke. These clusters exhibit proliferative potential, resembling germinal centers in spleens and lymph nodes. We then utilized a parabiosis model and confirmed that in situ proliferation, rather than peripheral infiltration, drives their formation. B lymphocyte ablation via CD20 monoclonal antibody improved ischemic injury and neuroinflammation, indicating the potential application of B lymphocyte–targeting therapies in ischemic stroke treatment. However, although there have already been studies demonstrating that B lymphocytes may be neurotoxic, with depletion of B lymphocytes alleviating ischemic injuries (24), it still remains controversial that one study has proposed that B lymphocytes promote neurogenesis and functional recovery at day 3 and day 7 after ischemic stroke (26). We suppose that this may be due to the differences in observation time point after ischemic stroke, the mechanisms of which potentially need to be further explored.

Microglia serve as the first-line responders to ischemic injury and interact with various CNS cell types primarily through the secretion of cytokines and chemokines (11). One study by Janelle et al. has shown that a subtype of B lymphocytes, the CD11b^hi^ B lymphocytes, accumulate in the ischemic lesions at day 7 after ischemic stroke, which contributes to both neuroinflammation and interactions between B lymphocytes and microglia (27). However, the interactions between microglia and B lymphocytes in the context of ischemic stroke have been insufficiently explored. In this study, we identified that microglia interact with B lymphocytes in ischemic stroke lesions in the later stage of ischemic stroke. Both CellChat analysis from scRNA-seq and Flowsight analysis indicate that this crosstalk may involve the MIF-CD74/CXCR4 signaling pathway.

MIF is an inflammation-related cytokine with chemokine-like functions, playing a role in various inflammatory responses within the CNS (28, 29). While MIF is known to regulate the activities of multiple cell types, including astrocytes, neurons, and oligodendrocytes (28), its role in modulating immune cell activities in the CNS following ischemic stroke remains underexplored. In our findings, both Cd74 and Cxcr4 expression levels were highest in B lymphocyte subcluster 2, which constitutes over half of the total B cells in the MCAO 7 day (7d) group. Simultaneously, MIF expression significantly increased in microglia from the MCAO 7d group, suggesting that the MIF-CD74/CXCR4 signaling pathway drives B lymphocyte recruitment by microglia during the early stages of ischemic stroke pathogenesis. Silencing MIF specifically in microglia not only reduced B lymphocyte infiltration and accumulation in the ischemic lesions but also ameliorated cerebral ischemic injuries.

IFN and their associated signaling pathways are typically linked to both infections and inflammation (30, 31). Previous studies have established the roles of IFNs in mediating the activities of various immune cells, including T lymphocytes, macrophages, and microglia (32–34). Specifically, under certain circumstances, such as chronic viral infection, IFN-related signature has been shown to regulate the phenotypes and functions of B lymphocytes (35). However, the roles of IFNs and related signaling pathways in ischemic stroke, particularly concerning B lymphocytes, remain to be fully elucidated. In our study, we found that B lymphocyte cluster 3, most associated with germinal center formation, exhibits high expression levels of IFN signaling pathway–related molecules, including Ifit1, Ifih1, and Ifit2. Blockade of the signals improved both ischemic injury and neuroinflammatory responses, especially microglia status and functions, suggesting that B lymphocytes may influence the CNS microenvironment after ischemic stroke via upregulating IFN-related signaling pathways, potentially related to influencing microglial activities. Thus, despite the potential discrepancies in murine and human pathology, targeting IFN-related signals could potentially alleviate ischemic injury and neuroinflammation, offering possible therapeutic avenues for ischemic stroke management.

In conclusion, our study demonstrates the massive accumulation of B lymphocytes within ischemic lesions, leading to the formation of follicle-like structures that exacerbate ischemic brain injury, which primarily results from in situ proliferation rather than recruitment from peripheral sources. Mechanistically, we identified the MIF-CD74/CXCR4 signaling pathway as a key mediator in the microglial recruitment of B lymphocytes following ischemic stroke. Additionally, IFN-related signaling pathways play crucial roles in the exacerbation of cerebral ischemic injury by B lymphocytes. Targeting these signaling pathways may offer promising therapeutic strategies against ischemic stroke, while more human studies are needed.

### Limitations of the current study.

The study still has some limitations. First, the human samples are from PBMCs of ischemic stroke patients, which may be distinctive from those from murine cerebral ischemic lesions. Firstly, lacking CSF and biopsy samples, as well as evidence of the clonal band, the link between human ischemic stroke and mouse MCAO models in B lymphocytes is relatively weak and needs more data to be validated (36). Second, proliferation could also occur in the peripheral B lymphocytes, although the number of proliferating B lymphocytes is relatively small. Also, there is a relatively high proportion of immature B lymphocytes in the dura, the functions and biological processes involved remain to be elucidated. In addition to mediating B lymphocyte chemotaxis, the MIF-CD74/CXCR4 signaling pathway could also potentially affect B lymphocyte cellular activities, as well as exert impacts on other cell types, which still remains to be further broadly explored.

## Methods

### Sex as a biological variable

Sex was not considered as a biological variable in this study. Male mice were used exclusively in this study.

### Animals

C57BL/6J male mice (WT; 20-25g; 8-10 weeks) were obtained from Hunan SJA Laboratory Animal Co. Ltd, Hunan, China. *Cx3cr1*^CreER^ mice [B6.129P2(Cg)-*Cx3cr1*^tm2.1(cre/ERT2)Litt/WganJ^] were obtained from The Jackson Laboratory. *Cd19*-IRES-DTRGFP mice (Cat. NO. NM-KI-190042) were purchased from Shanghai Model Organisms Center Inc. All mice were randomly divided into different treatment groups. All treatments and analyses were performed by investigators who are blinded to the design of the experiments.

### Experimental design overview

The experimental design was briefly described as follows. Generally, for WT mice in Figures 1 and 2, altogether 6 groups, including the sham-operated, post-MCAO 3d, 7d 14d, 28d, and 60d groups were analyzed, and 28d group was specifically studied for immunofluorescence and flow cytometry. Two samples of 28d post-MCAO group were used for spatial transcriptomics analysis in Figure 3. For parabiosis strategy in Figure 4, 4 pairs of parabionts were analyzed, and Sham-operated and 28d post-MCAO groups were utilized for further flow cytometry and immunofluorescence staining. For the CD20 B lymphocyte depletion experiment in Figure 5, 2 groups, including the Isotype group and CD20 mAb group were analyzed for behavioral tests, flow cytometry analysis, and immunofluorescence/immunohistochemistry analysis. Cells from 3 groups of mice, including the Sham-operated group, 7d post MCAO group and 28d post MCAO groups were utilized for scRNA-Seq analysis. For the microglial shMif silencing experiment (Figure 9), the NC group and shMif group post MCAO were utilized for further behavioral tests and immunofluorescence analysis. For the IFNAR blocking experiment (Figure 11), the Isotype group and IFNAR mAb group post MCAO were utilized for further immunofluorescence analysis. Data from Figures 12 and 13 include scRNA-Seq from 2 groups, including the healthy control and ischemic stroke patients.

#### Middle cerebral artery occlusion.

The MCAO model in mice was established as previously described with little modifications (37). Briefly, mice were anesthetized with isoflurane (3% for induction and 1.5% for maintenance), and after exposure of the common carotid artery and internal carotid, a monofilament with diameter of approximately 0.18 mm (L1800, Jialing Inc, Guangzhou) was used to block the cerebral blood flow in right hemisphere for 60 minutes. After that, the monofilament was removed to recover cerebral blood flow. Laser speckle contrast imaging was utilized to monitor cerebral blood flow throughout the process of modeling. Sham-operated mice received the same surgery procedure but without occlusion of the middle cerebral artery. Mice were excluded based on these criteria: (a) mouse did not show a greater than 75% CBF reduction or a less than 60% CBF reperfusion over baseline levels during MCAO surgery; (b) mouse that died during the surgery or before any behavioral tests; (c) no obvious infarction based on immunohistology analysis.

### Parabiosis

Parabiosis of WT mice and *Cd19*-DTR-eGFP transgenic mice were performed as previously described (38). First, a pair of mice (including 1 CD19-DTR-eGFP and 1 WT mouse with similar age and body weight) were housed in the same cage for 2 weeks before MCAO to get accustomed to each other. MCAO model was established in both the WT mice and transgenic mice, and 2 weeks after MCAO, parabiosis was performed between the post-MCAO WT mice and *Cd19*-DTR-eGFP transgenic mice. For parabiosis surgery, these 2 mice were placed in mirror image. Elbow and knee joints and the peritoneal cavities of both the 2 mice were sutured together. The parabionts were subcutaneously given antibiotics for possible infection, and 0.9% saline i.p. were also provided for hydration. After parabiosis, one single parabiont pair was housed in a single clean cage filled with bedding materials to help maintain sternal recumbency with the head up. Abundant moistened food pellets were provided on the cage floor to minimize the strain of reaching for food. In addition, the animals were continuously monitored for any signs of pain and hostile behavior towards each other. When any signs of attack behavior or hurt (such as continuous bleeding) occur, experiment is ended if necessary. Meanwhile, health and behavioral status of both mice were also evaluated by an experienced specialized veterinarian. Prophylactically, mice were treated with antibiotic oral suspension in their water bottle consecutively to prevent bacterial infections.

#### Behavioral tests.

Neurological deficits in mice were evaluated with modified neurological severity score (mNSS) as previously described (39). Briefly, this mNSS system is composed of motor abilities, reflex test and balance tests and the total score of neurological deficits ranges from 0 (completely no symptoms) to 14 (most severe). mNSS tests were conducted before surgery and 3d, 7d, 14d, and 28d after surgery.

### Tamoxifen injection

To induce the activity of Cre enzyme, 2-month old male *Cx3cr1*-Cre^+^ transgenic mice were intraperitoneally injected with tamoxifen (MedChemExpress, MCE; Cat #HY-13757A) once a day (100 mg/kg body weight, dissolved in corn oil at a final concentration of 20 mg/mL) for 5 consecutive days according to previous studies (40).

### Adeno-associated virus stereotactic injection

To specifically silence microglial *Mif* expression and further elucidate its role in ischemic stroke pathogenesis, stereotactic injection of adeno-associated virus was performed according to previous studies with some modifications (40, 41). Briefly, after induction of Cre activity by tamoxifen as previously described, mice were anesthetized with isoflurane and fixed in a stereotactic frame. The virus was stereotaxically injected into the right brain cortex (AP 0.02 mm, ML –3 mm, DV –2 mm). Total volume of the virus per mouse injected was 0.6–0.8 μL at a rate of 0.1 μL/min. After the end of the infusion, the syringe was not removed and was kept still for 10 min to allow the perfusion of the virus. 14 days after virus injection, MCAO surgery was implemented on mice, and 28 days after MCAO surgery, mice were anesthetized and euthanized, after which brain samples were harvested for further analysis.

### Flow cytometry analysis

Routine flow cytometry analysis was performed on CytoFlex (Beckman Coulter). Briefly, after transcardial perfusion with cold sterile PBS, ischemic brain hemispheres of MCAO mice and the hemispheres of sham-operated mice were harvested, placed in ice-cold tissue storage buffer (Miltenyi) and enzymatized with Enzyme P and Enzyme A for 30 minutes. Then, myelin debris were removed and cells pellets were resuspended with FACS buffer (PBS with 2% FBS), blocked with Fc block for 15 minutes, and stained with antibodies for 30 minutes on ice and protected from light. For dura tissues, cells from dura mater were isolated and prepared for flow cytometry as previously described with few modifications (15, 42). Briefly, animals were anesthetized with isofluorane and intracardially perfused with cold PBS. The skull cap was removed afterwards and the dura was peeled off under a binocular. Then, dura tissue was digested with both Collagenase D and Dnase I under 37°C for 45 minutes, followed by 1 volume of cell culture medium containing 10% FBS, followed by the same staining procedures as the brains. Right after the staining, the suspension was immediately utilized for flow cytometry analysis.

For experiments in Supplemental Figure 4, blood leukocytes were labeled by injecting fluorescence-coupled CD45iv intravenously into the tail vein of mice (Biolegend, Cat#147711, 3 μg per mouse). At 120 min after antibody injection, animals were anesthetized with isofluorane and intracardially perfused with cold PBS. Before perfusion, blood was taken and transferred in a collection tube. The cell suspensions for brain and dura tissues were prepared as described above.

#### FACS.

FACS was performed on Moflo XDP flow cytometry cell sorting (Beckman Coulter). The process of cell suspension preparation and cell staining was performed as described above. Viable 7-AAD^–^ CD45^+^ immune cells were used for subsequent single cell RNA-Sequencing analysis.

### Imaging flow cytometry analysis

Imaging flow cytometry analysis was performed on an Amnis Imaging Flow Cytometer (Millipore Sigma). Data analysis and figure illustration were performed by IDEAS software (Millipore Sigma).

### Single-cell RNA-seq analysis

10x Genomics high-throughput single-cell gene expression profiling library construction was utilized for generation of single-cell RNA-seq data. Briefly, cell suspension was assessed for cell viability. Then, the single-cell suspension was partitioned into GEMs (Gel Beads in Emulsions) and mRNAs are reverse transcribed into cDNAs. After processes including breaking GEMs, cDNA amplification, fragmentation, end repair and A-tailing, adaptor ligation, and PCR reactions system, products were then purified and went through library quality control (QC) and circularization. Then, in the sequencing step, single-stranded circle DNA molecules are replicated via rolling cycle amplification, and a DNA nanoball (DNB), which contain multiple copies of DNA, is generated. Sufficient quality DNBs are then loaded into patterned nanoarrays using high-intensity DNA nanochip technique and sequenced through combinatorial Probe-Anchor Synthesis (cPAS).

Seurat package (v 4.04) (43) was used for subsequent analysis. Cells that express fewer than 200 genes were excluded, and cells with greater than 90% of the maximum genes were also discarded to avoid ‘doublet’ events. In order to prevent mitochondrial contamination, we identified cells with greater than 7.5% mitochondrial genes as poor-quality cells and excluded. The expression matrix was normalized using the normalizeData function and the top 2,000 variable genes were calculated with FindVariableFeatures. The Seurat object was then scaled with the scaleData function and principal component analysis (PCA) was further implemented. The top 20 principal components were implemented for the unsupervised clustering. ‘FindIntegrationAnchors’ was utilized to remove batch effects among different samples. FindNeighbors function was used to construct the *k*-nearest neighbors graph, and FindClusters function was used to iteratively group nuclei (resolution = 0.5). The cell type of each cluster was identified based on the cellular markers identified based on FindAllMarkers function. For the reclustering of microglia/macrophages, the top 10 principal components were used for the clustering. R package monocle (2.20.0) (44), clusterProfiler (v 4.0.5) (45), msigdbr (7.4.1), ggplot2 (v 3.3.5), and Pheatmap (1.0.12) were utilized for subsequent analysis. For RNAVelocity analysis, Python (3.9.21), scvelo (0.3.3), and velocyto (0.17.17) are utilized.

#### Ingenuity pathway analysis.

IPA was implemented according to the manufacturer’s instructions. In this paper, we’ve mainly focused on Canonical Pathways in core analysis functions, and pathways with *P* < 0.05 were considered significant.

#### Immunofluorescence staining.

Immunofluorescence staining was performed as previously described (46). Briefly, mice brain sections were incubated with primary antibodies overnight at 4°C, and then incubated with secondary antibodies at room temperature for 1 hour in the dark. Images were obtained with a confocal microscope (OLYMPUS, FV1200). The border of infarction was determined by the morphology of NeuN^+^ (Cell Signaling Technology, Cat#12943) neuron and Iba-1^+^ (WAKO, Cat#019-19741) microglia, and the periinfarct area was defined as the tissue that covers a radial distance of 200–300 μm from the border of the infarct, as previously reported (47). Morphological analysis regarding cell surface area, volume, and sphericity was implemented with Imaris software and according to previous studies (48). For each mouse, 4–5 microscopic fields were captured and the average values were utilized for statistics analysis. Antibodies and reagents used in this study are included in Supplemental Table 10.

### Demyelination quantification analysis

Area of demyelination quantified by LFB staining was performed as previously described (47). Briefly, brain sections were stained with LFB dye (G1030, Servicebio) at 60°C for 6–8 hours. Sections were then differentiated alternately in 0.05% lithium carbonate solution and 70% ethanol, then dehydrated stepwise with 75%, 90%, and 100% ethanol. Finally, sections were soaked in xylene for 5–10 min and sealed with neutral resin. Images were captured by a microscope (BX51, Olympus, Japan). The area of demyelination in corpus callosum were calculated by using ImageJ (NIH).

The regions of interest (ROI) were picked at the same approximate location across different brain slices at the infarction borders. Myelin staining was measured by positive pixel identification using the same threshold cutoff in ImageJ across animals. To generate a percentage, (the total area minus positive-stained area) was divided by the total area of the ROI, which serves as the percentage of demyelination.

### Spatial Mip-seq analysis

The specific targeting probes were designed by Spatial FISH Ltd. Samples were fixed by 4% paraformaldehyde, then covered with reaction chamber to perform the following reactions. After dehydration and denaturation of samples with methanol, the hybridization buffer with specific targeting probes was added to the chamber for incubation at 37°C overnight. Then, samples were washed three times with PBST, followed by ligation of targeting probes in ligation mix at 25°C for 3 hours. Next, samples were washed 3 times with PBST and subjected to rolling circle amplification by Phi29 DNA polymerase at 30°C overnight. Subsequently, the fluorescence detection probes in hybridization buffer were applied to samples. Finally, samples were dehydrated with an ethanol series and mounted with mounting medium. After capturing images by Leica THUNDER Imaging Systems, 20× (NA = 0.80), signal dots were decoded to interpret RNA spatial position information.

### Spatial transcriptomics analysis

The Visium HD Spatial Gene Expression Platform (10x Genomics) achieves single-cell-level spatial resolution with 2 × 2 μm barcoded squares without gaps.

Fresh frozen OCT-embedded tissues (FF tissues) were assessed by RNA integrity quality using RIN score. And the tissue blocks were also assessed by tissue morphology through DAPI and H&E staining. For the high RNA quality, RIN≥4 was required.

According to the “Visium HD Fresh Frozen Tissue Preparation Handbook” (CG000763), a 10-μm-thick section of FF tissue was placed on a histology slide and stored at –80°C until it was ready for spatial expression. Subsequently, the tissue was stained with hematoxylin eosin (HE) and imaging. Probe hybridization, probe ligation, slide preparation, probe release, extension, library construction were performed according to the “Visium HD Spatial Gene Expression Reagent Kits User Guide” (CG000685). The libraries were then sequenced on Illumina NovaSeq X plus with paired-end reads, performed by LC-Bio Technology Co., Ltd, Hangzhou.

### Intracerebroventricular injection

Anti-mouse IFNAR antibody was injected to block IFN-related receptor activities in MCAO mice. The intracerebroventricular injection of anti-IFNAR antibody (BioXCell, Cat#BE0241) was performed as previously described. Briefly, neutralizing antibody to mouse IFNAR1 or its corresponding isotype control were injected i.c.v. (0.4 mm posterior to the bregma, 1.0 mm lateral to the midline and 2.0 mm in depth from the brain surface) at a dose of 10 μg per mouse (38, 49).

### CD20 monoclonal antibody injection strategy

CD20 monoclonal antibody (BioXCell, Cat#BE0356) was intraperitoneally injected into mice to ablate B cells as previously described with little modifications (50). Briefly, 100 μg CD20 mAb was injected intraperitoneally into WT mice 24 hours prior to MCAO surgery. After MCAO, the same dose of CD20 mAb was given to mice every 10 days. Both flow cytometry and immunofluorescence analysis were utilized to examine the efficiency of B lymphocyte ablation by CD20 mAb.

#### Primary microglia culture.

Primary mouse microglia were extracted from brains of 0–3 days postnatal mouse pups and cultured as previously described (46). Briefly, the brains of the P 0–3 neonatal mice were cut up and digested with 0.125% trypsin. The isolated cells were cultured in poly-d-lysine–coated flask with DMEM/F12 containing 10% heat-inactivated FBS. Then the cells were cultured in a humidified incubator at 37°C with 5% CO_2_ for 10–12 days. After that, microglia at the upper layer were harvested for further treatment.

### Murine B cell sorting and culture strategy

Murine B cells were sorted by MACS and subsequently cultured based on manufacturer’s instructions (B cell isolation kit, Miltenyi) and established protocols (51, 52). Briefly, mice were anesthetized with isofluorane, then sacrificed and the spleens were taken from the abdominal cavity. Splenocytes were treated with ACK solution to remove erythrocytes. The remaining leukocytes were incubated with 10 μl cocktail antibody, followed by 20 μl anti-biotin magnetic bead-conjugated antibody (Miltenyi, Cat#130-090-862) at 4°C for 15 minutes each. The antibody-incubated leukocytes were added onto the prerinsed LS column, and the flow-through contains purified B cells and was collected in a centrifuge tube.

The sorted B cells were cultured in RPMI-1640 cell culture medium added with anti-CD40 antibody (BioXCell, Cat#BE0016-2, 10 μg/mL), anti-IgM antibody (Biolegend, Cat#157102, 10 μg/mL) and R848 (MedChemExpress, Cat#HY-13740, 0.05 μM) for 3 consecutive days. These stimulated B cells were then utilized for subsequent experiments.

#### Oxygen-glucose deprivation-reperfusion (OGD/R).

Oxygen-glucose deprivation-reperfusion model in primary mouse microglia in vitro was conducted as previously described (53). Briefly, OGD was performed with medium replaced with no-serum PBS and incubated in a non-CO_2_ incubator for 4 hours at 1% O_2_ concentration. Reperfusion was performed with DMEM/high glucose medium containing 20% FBS and incubation in a normal incubator containing 20% O_2_ and 5% CO_2_ for 24 hours.

#### Seahorse metabolic analysis.

Seahorse metabolic assays were conducted as previously described (37). Briefly, primary mouse microglia were seeded into XFe24 cell culture plates at a density of 80,000 cells per well. Mitostress kit (Agilent, Cat#103015-100) and glycolytic rate assay kit (Agilent, Cat#103344-100) was used to measure oxygen consumption rates. Cell culture medium was replaced with fresh running buffer (XF DMEM plus 10 mM glucose, 2 mM glutamine, and 1 mM pyruvate) and incubated in a non-CO_2_ incubator for 60 minutes. 1.5 μM oligomycin, 2 μM FCCP, and 0.5 μM rotenone plus antimycin A were added to the sensor cartridges in calibration processes. For glycolytic rate assay, rotenone plus antimycin (0.5 μmol/L) and 2-DG (50 mmol/L) were utilized. Values of OCR and glycoPER were normalized to the cell number in each well. The values of the first measurement points of OCR for WT-control or negative control–transfected microglia were normalized to 1 according to previous study (54).

#### RNA extraction and real-time PCR.

Total RNA from primary microglia or MACS-sorted microglia were extracted by TRIzol following manufacturer’s instructions. Then, cDNA was reversely transcribed from 1 μg of RNA using PrimeScript RT Master Mix (TAKARA, RR036A). A total of 20 μL reaction system was prepared for quantitative RT-PCR using Hieff qPCR SYBR Green Master Mix (YEASEN, 11201ES03).

Real-time PCR system (CFX96, Bio-Rad) was used for all reactions. The expression levels of target genes were normalized to β-actin and calculated using 2ΔΔ^Ct^ method. Primers used in this study are included in Supplemental Table 11.

#### siRNA transfection.

Mouse siRNA and negative control were purchased from Obio Tech (Shanghai, China). The whole process of transfection was conducted according to manufacturer’s protocol (Lipofectamine RNAiMAX, ThermoFisher, Cat#13778030). Briefly, we mixed the diluted siRNA and Lipofectamine RNAiMAX at a ratio of 1:1 and incubated for 5 minutes, then added the siRNA-lipid complex to cells and incubated for 24 hours. After that, the transfected cells were utilized for further experiments.

### Human PBMC collection strategy

All of the experiments using human samples were approved by the ethics committee of Tongji Medical College, Huazhong University of Science and Technology (HUST). PBMCs from both 4 ischemic stroke patients (2 males and 2 females) and 4 age- and sex-matched healthy control (2 males and 2 females) and were isolated and collected as previously described for scRNA-Seq analysis. In this study, the patients were in the recovery stage of cerebral infarction (greater than 10 days after the onset of cerebral ischemic stroke). Briefly, the clinical diagnosis of acute ischemic stroke was confirmed by cranial magnetic resonance (MR) examinations with diffusion weighted imaging (DWI), and intracranial large vessel status was evaluated by digital subtraction angiography (DSA) in every patient. The presence of intracranial carotid artery terminus or first middle cerebral artery segment (M1) occlusion was confirmed on DSA. Healthy volunteers with no prior history of stroke were matched with patients with ischemic stroke for age and sex, and were recruited as a healthy control group. Cranial MR with DWI and angiography were performed in healthy volunteers to verify that no silent brain infarcts or artery stenosis were present. Age and sex for the patients and healthy controls were shown in Supplemental Table 9.

Briefly, 4 mL freshly collected blood sample were diluted with 1:1 volume of HBSS/DPBS and slowly added to the Ficoll layer in the 15 mL centrifugation tube. After centrifugation at 500*g*, 20°C for 20 minutes, with no brake, we collected the interface layer and diluted it with 5 times volume of HBSS/DPBS. We resuspended the cell pellet with previously prepared cell freezing solution (FBS+DMSO, with ratio of 9:1) and placed the tube into a gradient freezing box and stored the box in the –80°C refrigerator before cell sorting. Human CD19^+^ B lymphocytes were subsequently sorted using FACS as previously described and used for further analysis.

### Statistics

Data in the figures are presented in the form of mean ± SD, and normalization and transformation methods are shown in respective figure legends. The number of animals and In vitro replicates for each experiment in different groups are also shown in the figure legends. For most animal studies, *N* = 6 per group is utilized based on previous studies (47). Unpaired 2-tailed *t* test and 1-way or 2-way ANOVA followed by Bonferroni test were performed by GraphPad Prism software version 8, and in a few cases, nonparametric Mann-Whitney test and Kruskal-Wallis test were performed. *P* value < 0.05 was considered statistically significant.

### Study approval

All animal surgeries were approved by the Animal Care Committee of Tongji Medical College, Huazhong University of Science and Technology. For collection of human PBMC samples, the collection of all clinical samples in this study was approved by the Ethics Committee of Tongji Hospital, Tongji Medical College, Huazhong University of Science and Technology. All participants provided written informed consent.

### Data availability

The data for RNA-seq in this paper are deposited into NCBI SRA database with the accession number PRJNA1337929. All data in this paper are available from the corresponding authors upon reasonable request.

## Author contributions

DST, WW, CQ, SY and HZ conceptualized and designed the study. CQ, MHD, YHC, LQZ, HZ and SY analyzed data. WW, DST, CQ, MHD, LQZ, LYZ and XWP interpreted data. SY and HZ drafted the manuscript. WW, DST and CQ made critical revision of the manuscript. SY, HZ, LLX, LQZ, YHC, LC, XWP, LYZ, LFZ, MHD, KS, JX, LJW, WW, DST, and CQ were involved in collection and critical review of the data. All authors approved the final version of the manuscript.

## Funding support

- Ministry of Science and Technology China Brain Initiative Grant (STI2030-Major Projects 2022ZD0204700 WW).
- National Natural Science Foundation of China (Grants: 82371404 DST, 82271341 CQ, 81873743 DST, 82401565 SY).
- Key Research and Development Program of Hubei Provincial Department of Science and Technology (2023BCB148).

## Supplementary Material

Supplemental data

Supplemental table 1

Supplemental table 2

Supplemental table 3

Supplemental table 4

Supplemental table 5

Supplemental table 6

Supplemental table 7

Supplemental table 8

Supplemental table 9

Supplemental table 11

Supporting data values

## Version 1 03/02/2026

Electronic publication
