# Supplementary material for: Ectopic B lymphocyte follicles exacerbate ischemic brain damage via MIF-CD74/CXCR4 and interferon signaling
Source: J Clin Invest. 2026 Mar 2;136(5):e196905. doi: 10.1172/JCI196905 (PMC12948440; doi:10.1172/JCI196905)
Supplement: Supplemental data [file jci-136-196905-s239.pdf]

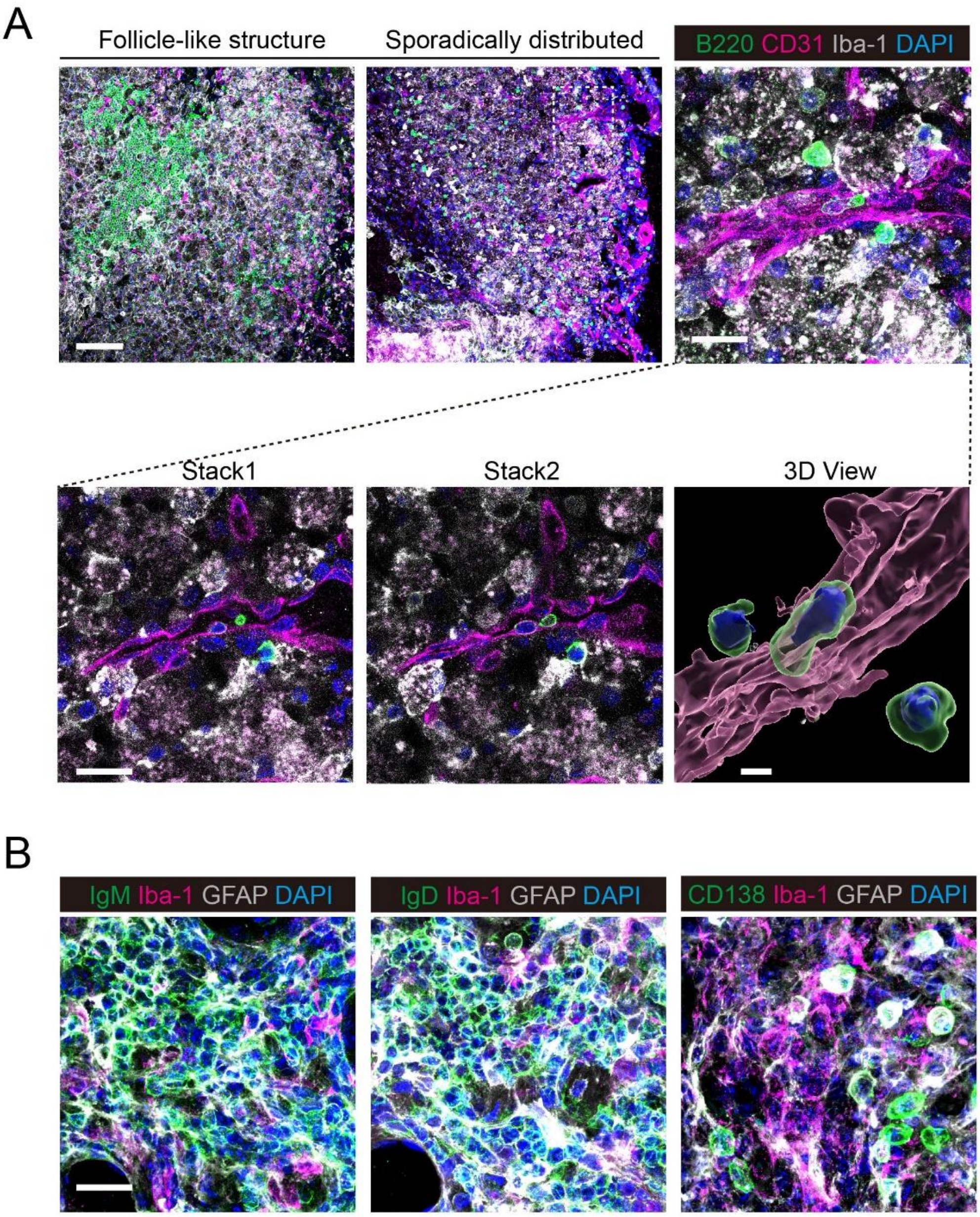

Supplementary Figure 1. The position of B lymphocytes and spatial relationship with other cells in the cerebral ischemic lesions.

A: Colocalization of B lymphocytes with CD31+ cerebral microvessels, Scale Bar: 100um for the low magnification, 20um for high magnification and 5um for the imaris 3D-reconstruction illustration;

B: Colocalization of B lymphocytes with GFAP+ astrocytes and Iba-1+ microglia in the ischemic lesions, Scale Bar: 20um.

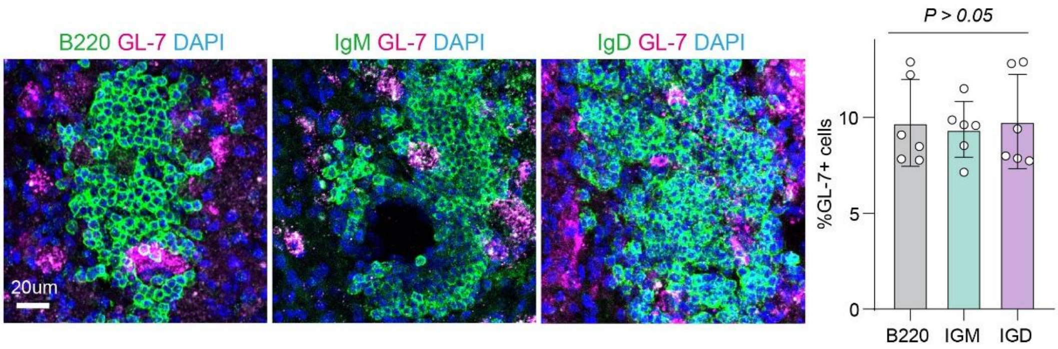

Supplementary Figure 2. Proportions of GL7+ cells in different B cell subclusters.

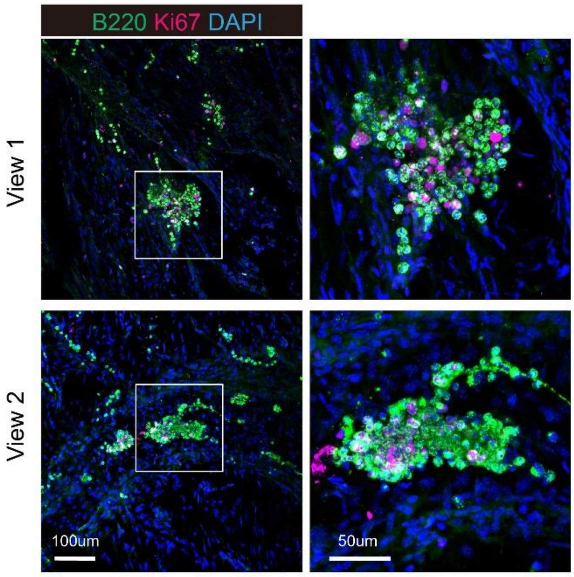

Supplementary Figure 3. B cell aggregates with Ki67+ in dura of mice after MCAO

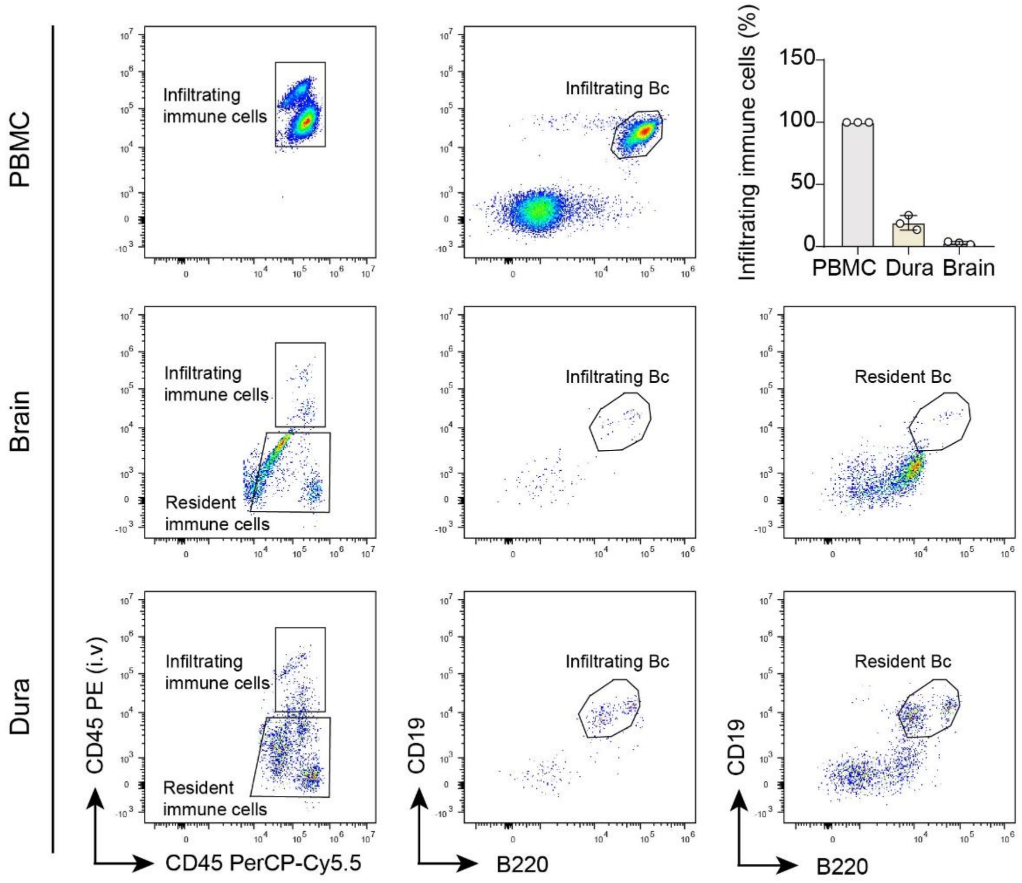

Supplementary Figure 4. Illustration of infiltrating immune cells and resident immune cells in the PBMC, brain and dura of sham-operated mice by flowcytometry.

CD45-PE antibody was intravenously injected into the tail vein of mice (Biolegend, 3 μg per mouse) to distinguish peripherally infiltrated immune cells (CD45-PE positive) and resident immune cells (CD45-PE negative). At 120 min after antibody injection, animals were anesthetized with isofluorane and intracardially perfused with cold PBS. Before perfusion, blood was taken and transferred in a collection tube. The cell suspensions for brain and dura tissues were prepared as described above. Percentages of peripherally infiltrated immune cells was also shown. N = 3 biological replicates per group.

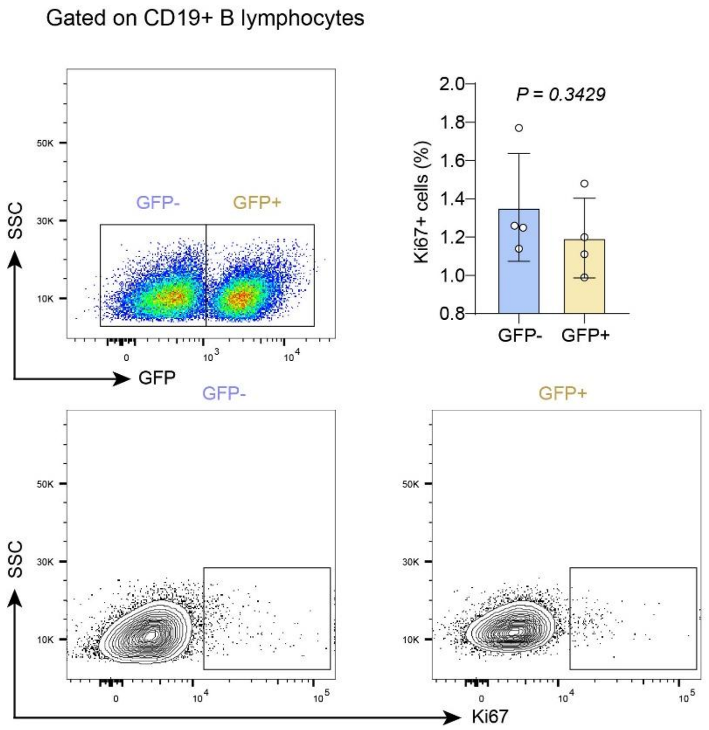

Supplementary Figure 5. Proportion of Ki67+ B lymphocytes in both the GFP- and GFP+ subclusters in PBMCs after parabiosis between WT mice and *Cd19*-DTR-GFP mice.

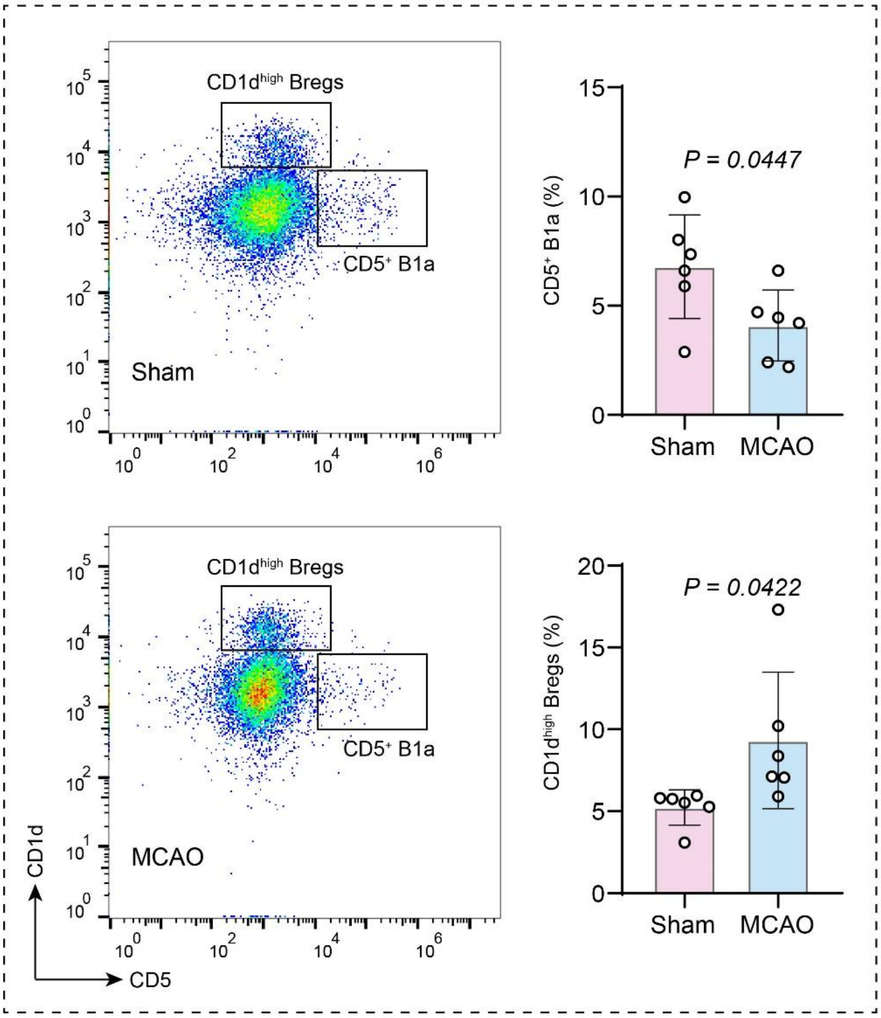

Supplementary Figure 6. Comparison of proportion of Bregs and CD5+B1a cells by flowcytometry.

CD1d and CD5 antibodies were utilized to mark Bregs and B1a cells in the spleen. Comparisons of the proportions of these two types of cells between Sham and MCAO groups are performed. N =6 per group.

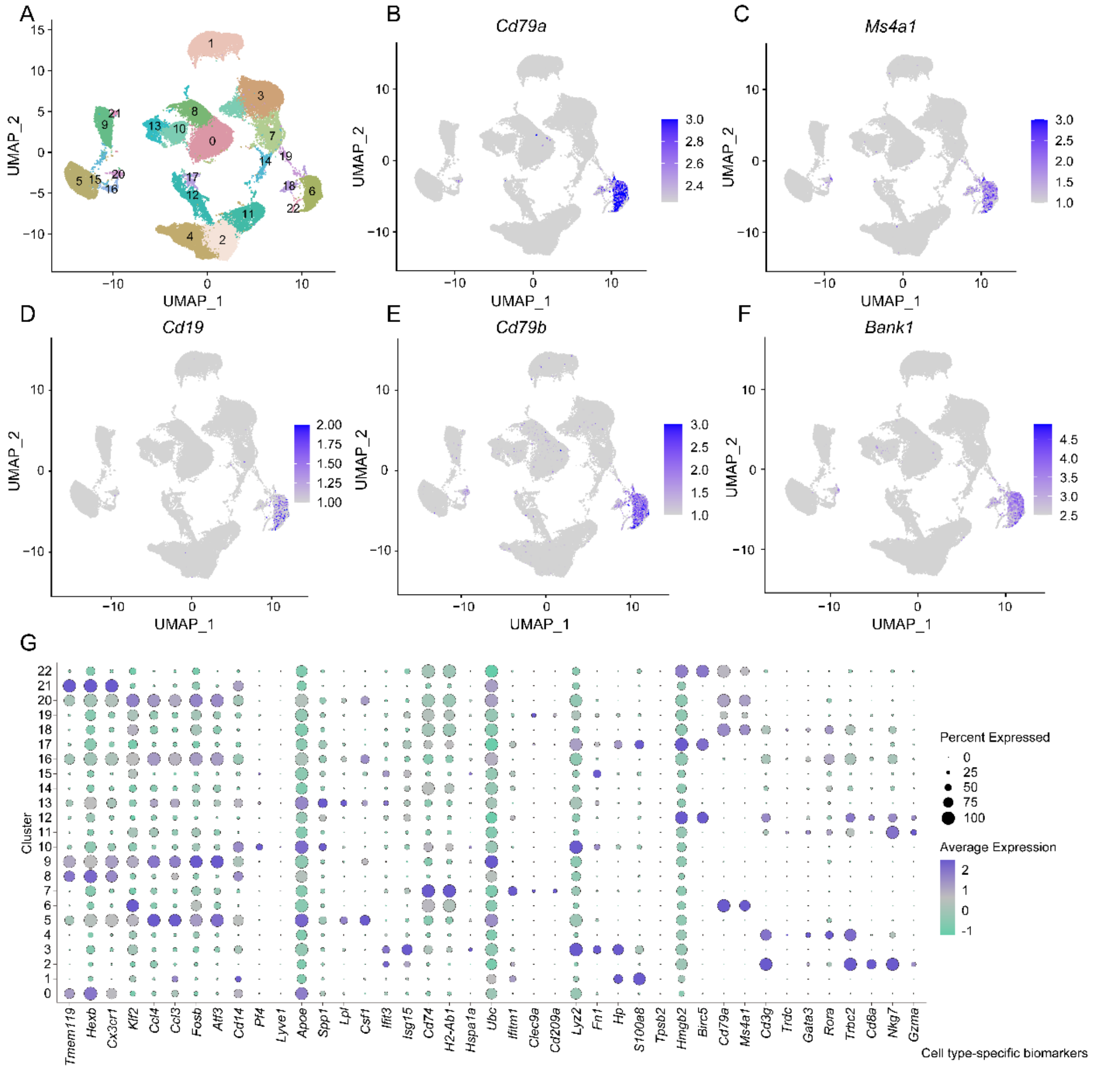

Supplementary Figure 7. scRNA-Seq analysis of the CD45+ immune cells in the brains of mice.

A: UMAP plot illustrating different clusters of the CD45+ immune cells in the brains of mice.

B: Expression levels of Cd79a in the immune cells, illustrated by UMAP plot.

C: Expression levels of Ms4a1 in the immune cells, illustrated by UMAP plot.

D: Expression levels of Cd19 in the immune cells, illustrated by UMAP plot.

E: Expression levels of Cd79b in the immune cells, illustrated by UMAP plot.

F: Expression levels of Bank1 in the immune cells, illustrated by UMAP plot.

G: Expression levels of different representative biomarkers in all the immune cells to define the cells types of these CD45+ immune cells, illustrated by bubble plot.

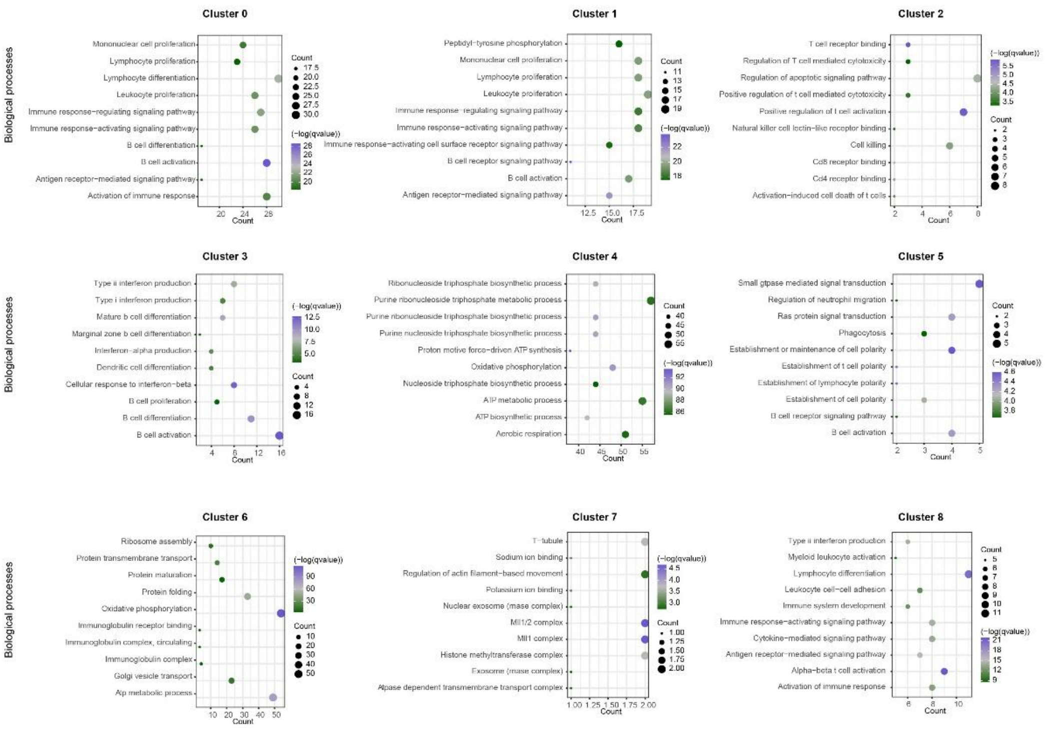

Supplementary Figure 8. Biological processes enriched for each B lymphocyte subclusters from the scRNA-Seq dataset.

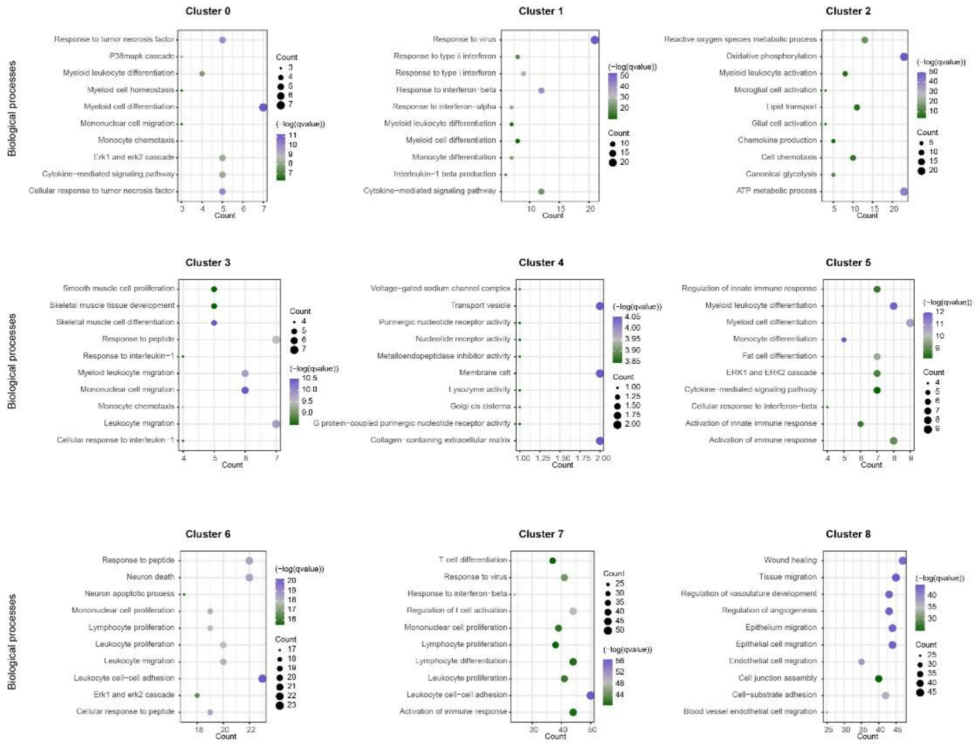

Supplementary Figure 9. Biological processes enriched for each microglia subcluster from the scRNA-Seq dataset.

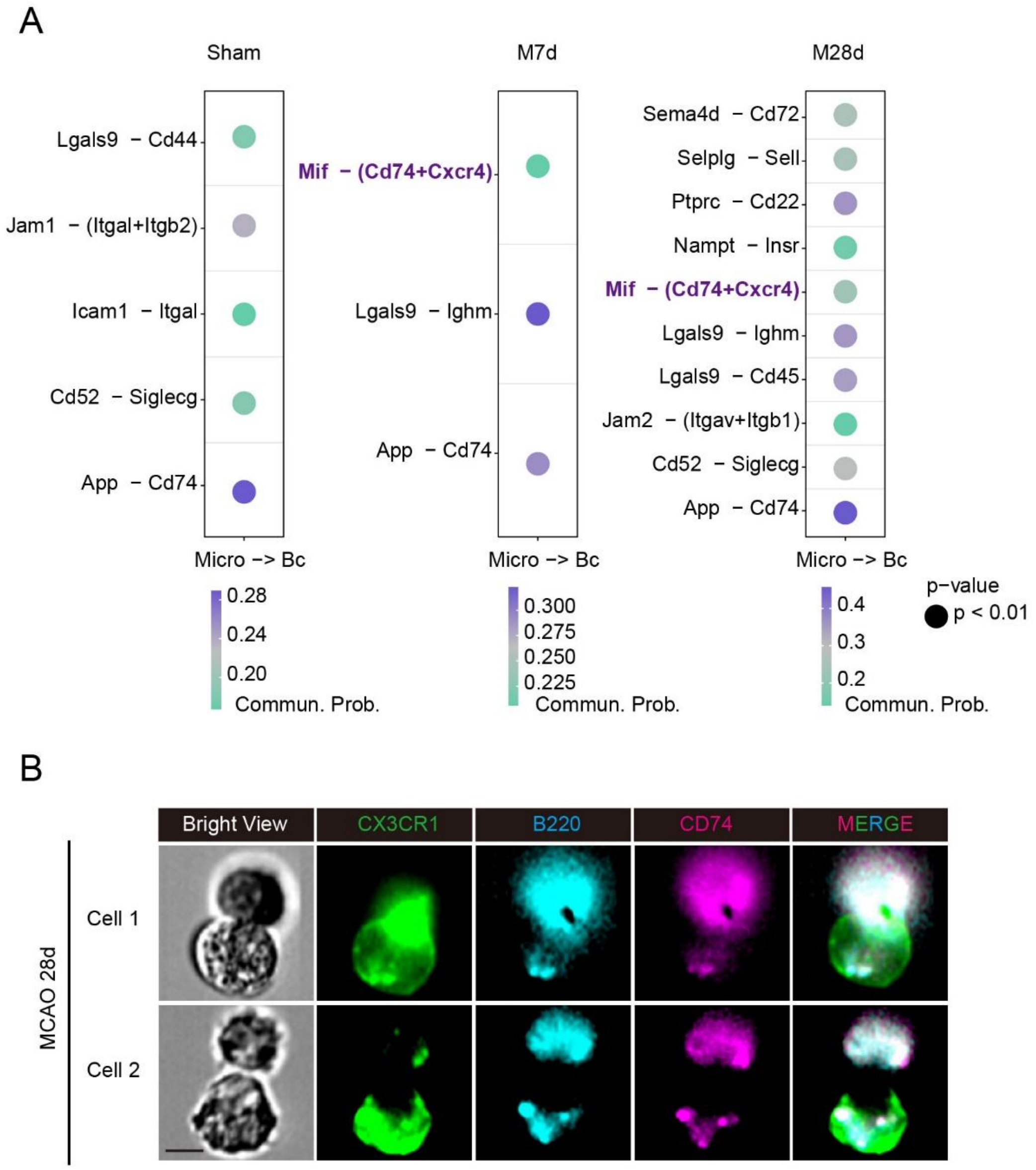

Supplementary Figure 10. MIF-CD74/CXCR4 signaling pathway mediates the interactions between B lymphocytes and microglia in ischemic stroke.

A: Bubble plot showing the signaling pathways involved in the cell-cell interactions between B lymphocytes and microglia at different time points post ischemic stroke;

B: Relationship between microglia and B lymphocytes in the ischemic lesions, with expression of CD74 highlighted, Scale Bar: 5um.

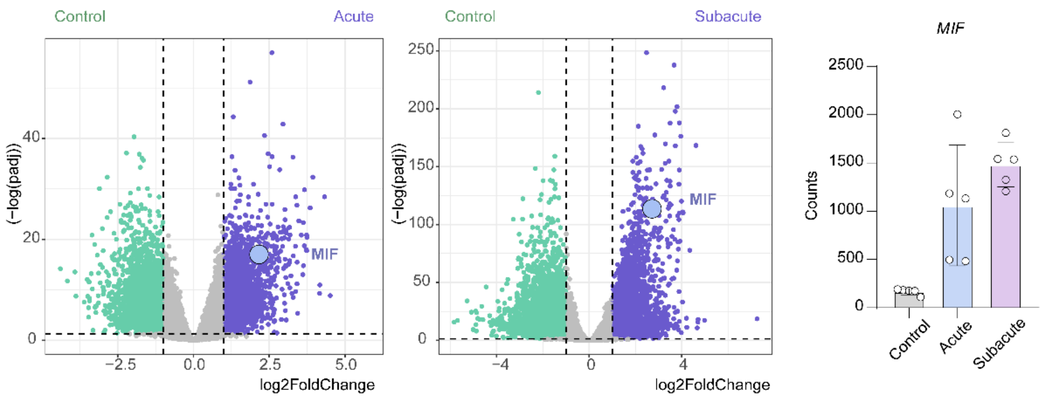

Supplementary Figure 11. Expression levels of MIF in PBMCs of Control, Acute and Subacute ischemic stroke patients, using public data from Gene Expression Omnibus (GEO). Expression of MIF and differentially expressed genes analysis between Acute and Control, Subacute and Control patients, respectively, illustrated by volcano plot. Expression levels of MIF were also illustrated by bar plot.

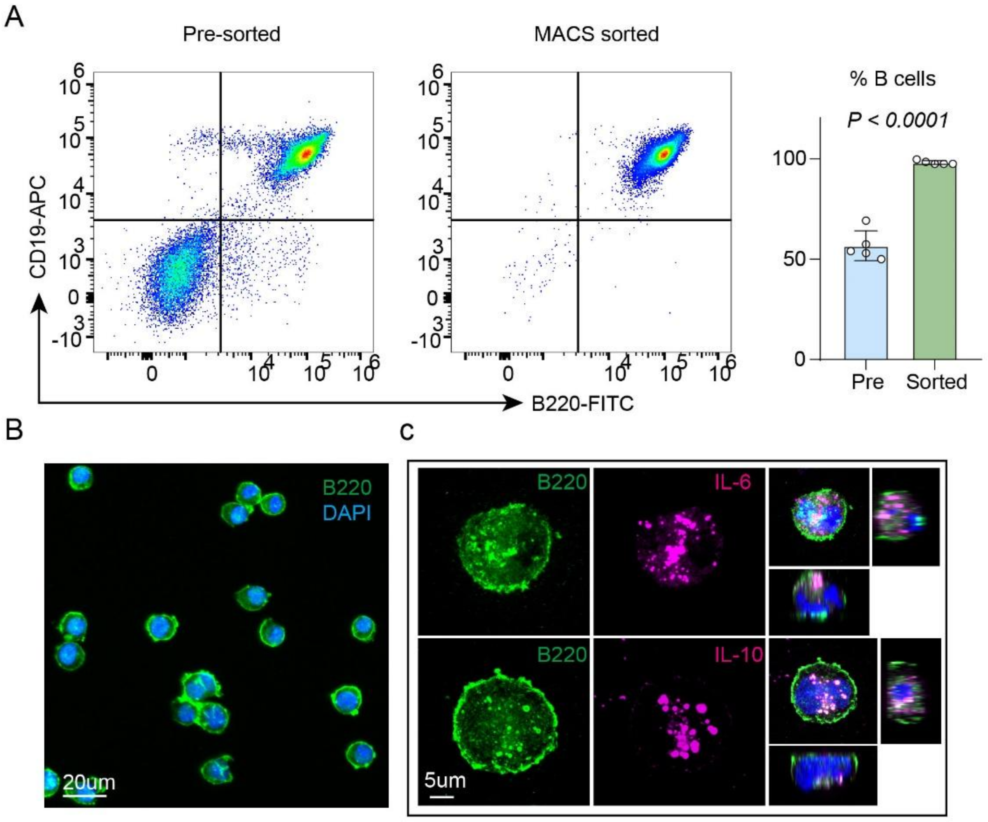

Supplementary Figure 12. Purification of murine B lymphocytes and validation of its purity. A: Validation of the purity of MACS-sorted B lymphocytes by flowcytometry analysis;

B: Validation of the purity of MACS-sorted B lymphocytes by immunofluorescence, with the pro-inflammatory and anti-inflammatory phenotypes illustrated, Scale Bar: 20um and 5um, respectively.

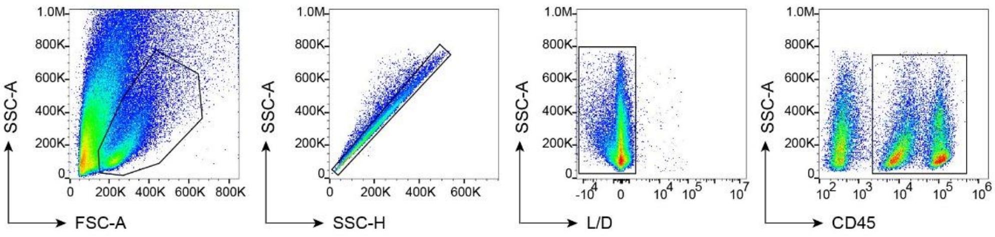

Supplementary Figure 13. FACS sorting strategy of murine brain CD45+ cells for scRNA-Sequencing.

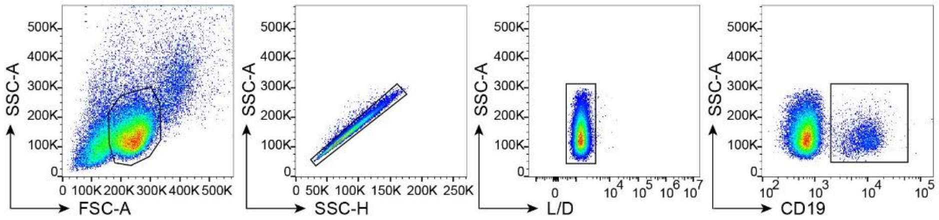

Supplementary Figure 14. FACS sorting strategy of human CD19+ cells for scRNA-Sequencing.

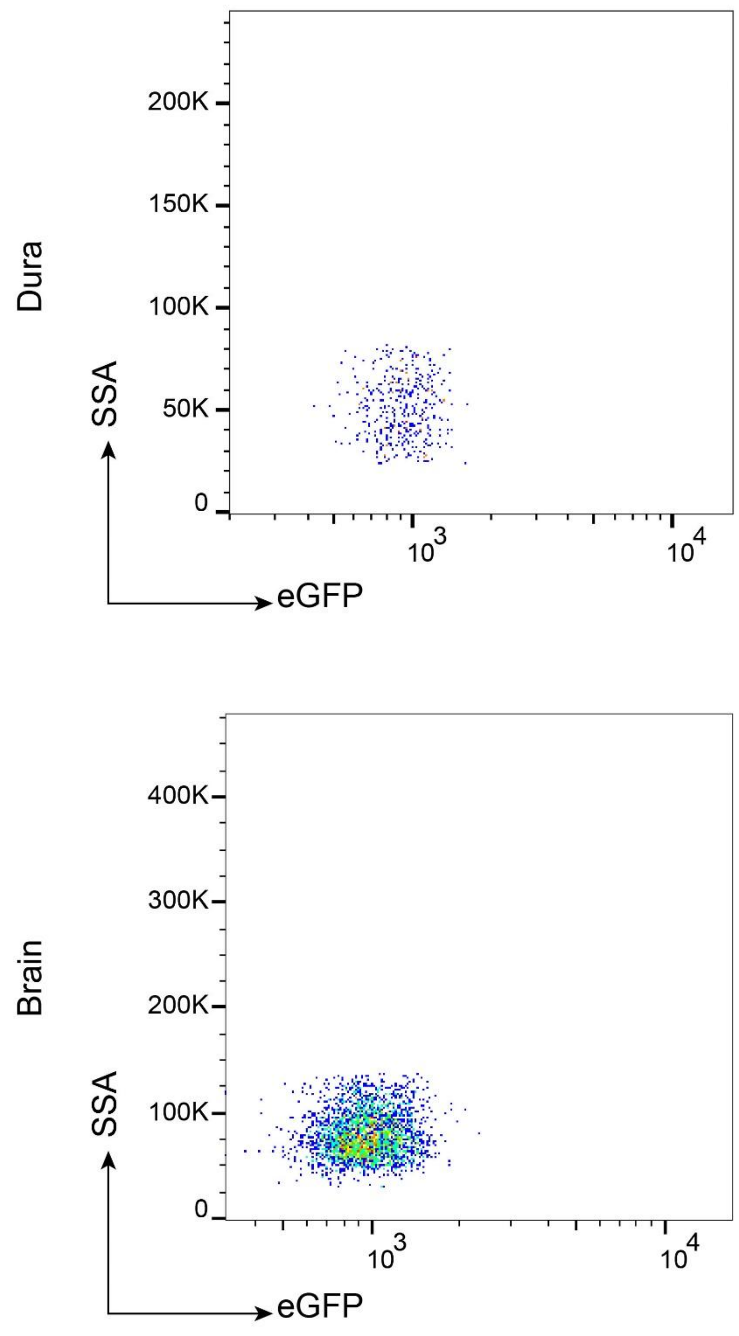

Supplementary Figure 15. FMO controls (staining miuus GFP) for dura (above) and brain (below).

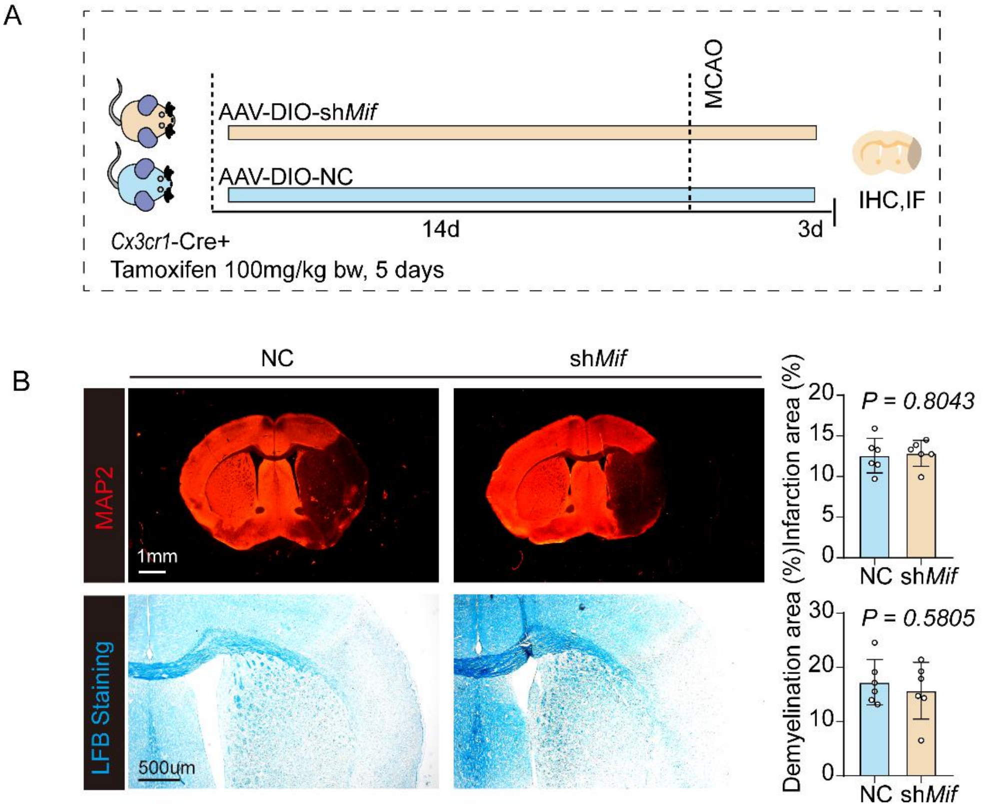

Supplementary Figure 16. Effects of silencing microglial Mif on cerebral ischemia at 3 days post ischemic stroke. A. Schematic illustrator indicating the study design of this part of experiment. B. Effects of silencing microglial Mif on infarction area (MAP2 staining) and demyelination area (LFB staining). N = 6 per group.

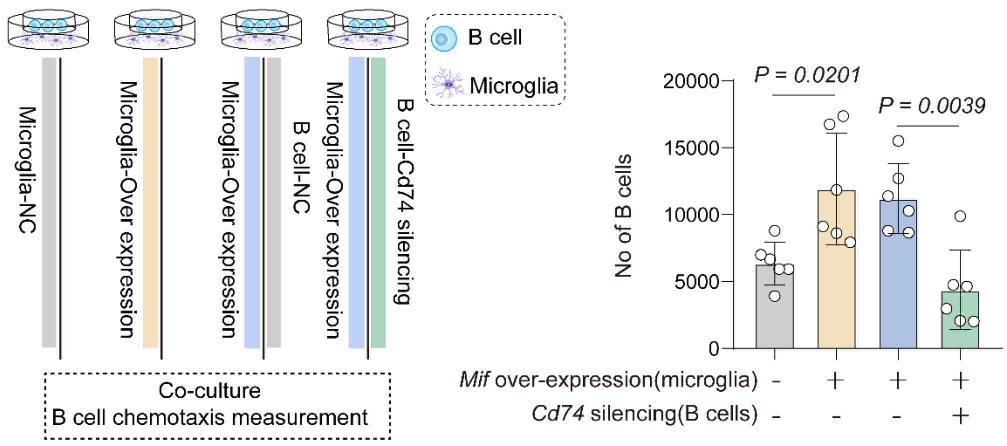

Supplementary Figure 17. Effects of Mif-Cd74/Cxcr4 signaling pathway on B lymphocyte chemotaxis induced by microglia *in vitro*.

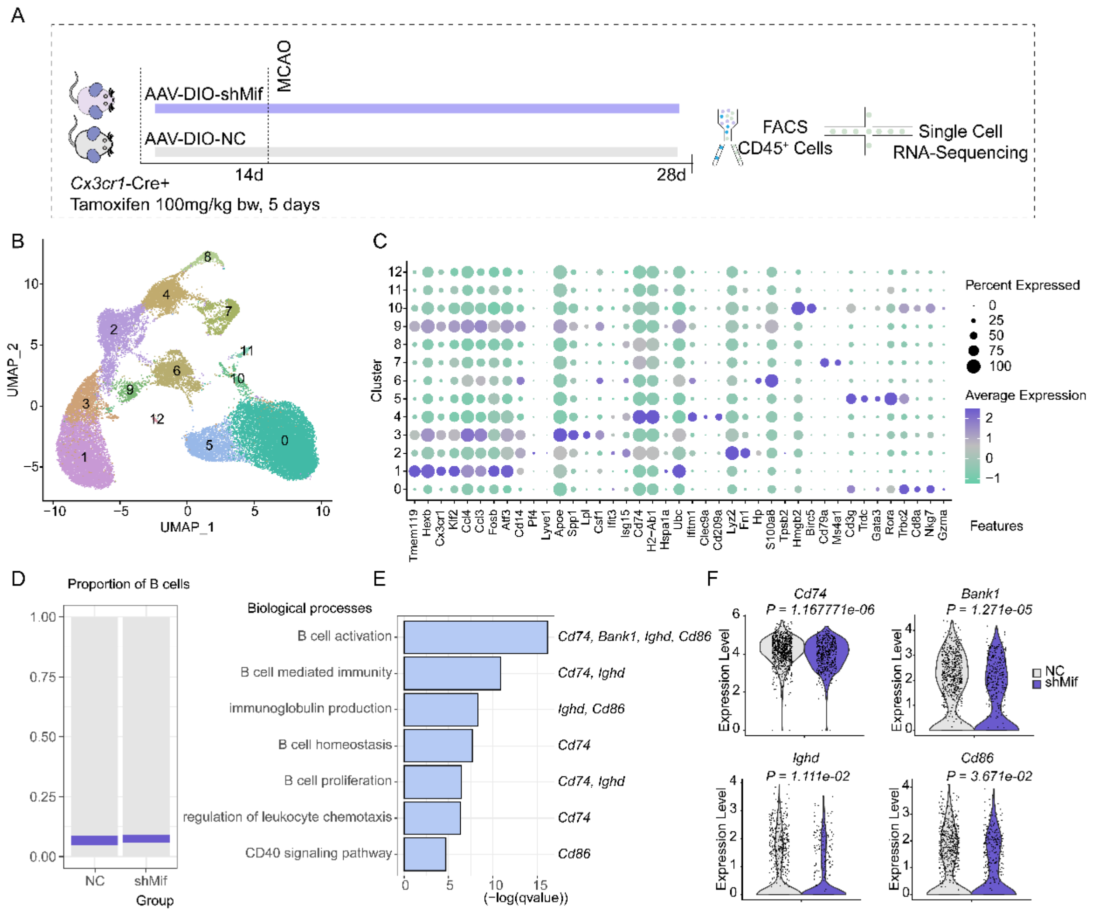

Supplementary Figure 18. Effects of silencing microglial Mif on cerebral ischemia at 28 days post ischemic stroke. A. Schematic illustrator indicating the study design of this part of experiment. B. UMAP clustering of different cell types. C. DotPlot showing the representative biomarkers for different cell types of the scRNA-Seq dataset. D. Proportions of B lymphocytes in both two groups. E. Biological processes enriched for the feature genes of B lymphocyte, in which activities related to B cells are involved. F. Expression levels of Cd74 and the other B cell-activity related biomarkers, including Bank1, Ighd and Cd86 in both two groups.

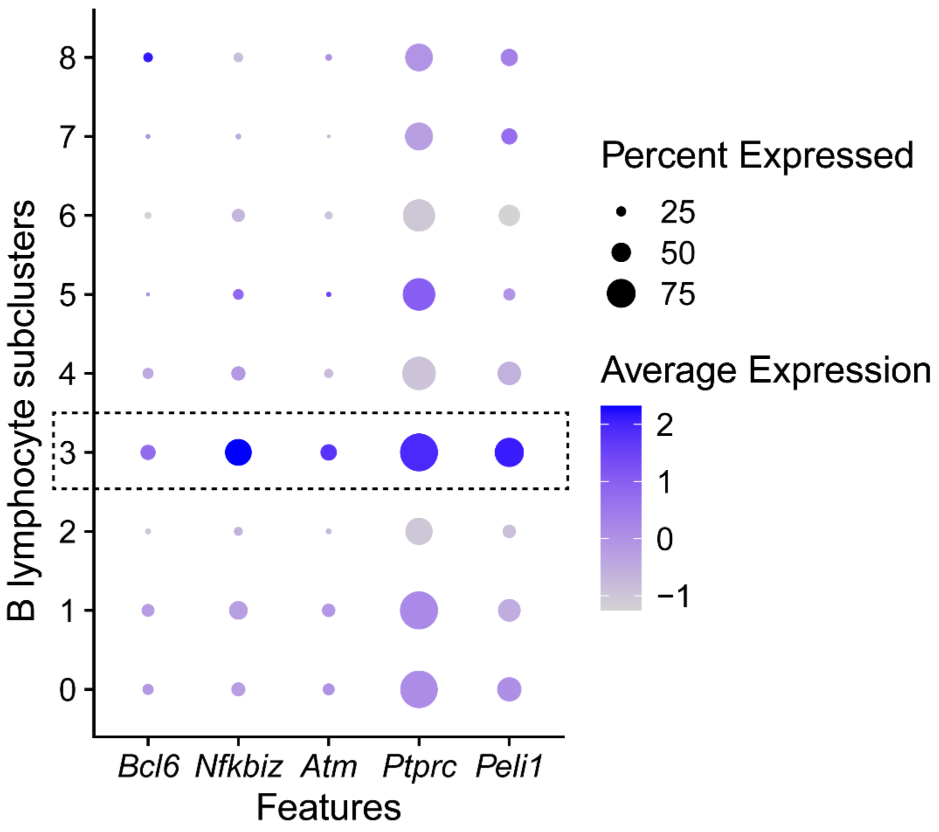

Supplementary Figure 19. Expression levels of germinal center formation-related molecules in different B lymphocyte subcluster.

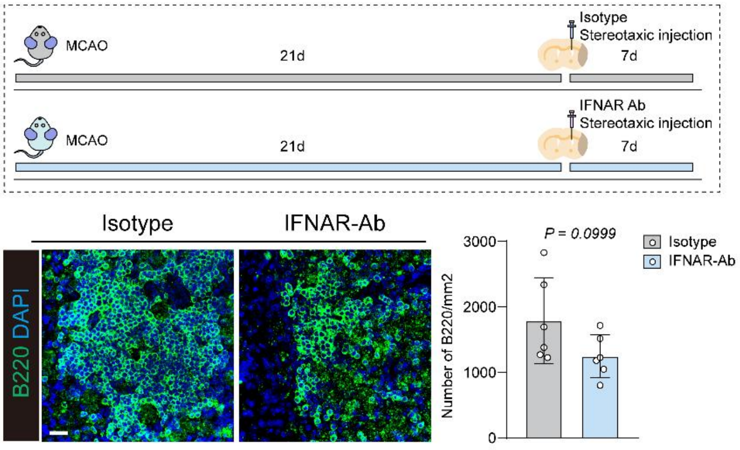

Supplementary Figure 20. Effects of blocking IFNAR on the formation of B cell follicle-like structures post ischemic stroke. A. Schematic illustrator indicating the study design of this part of experiment. B. Effects of s blocking IFNAR on the formation of B cell follicle-like structures. N = 6 per group.

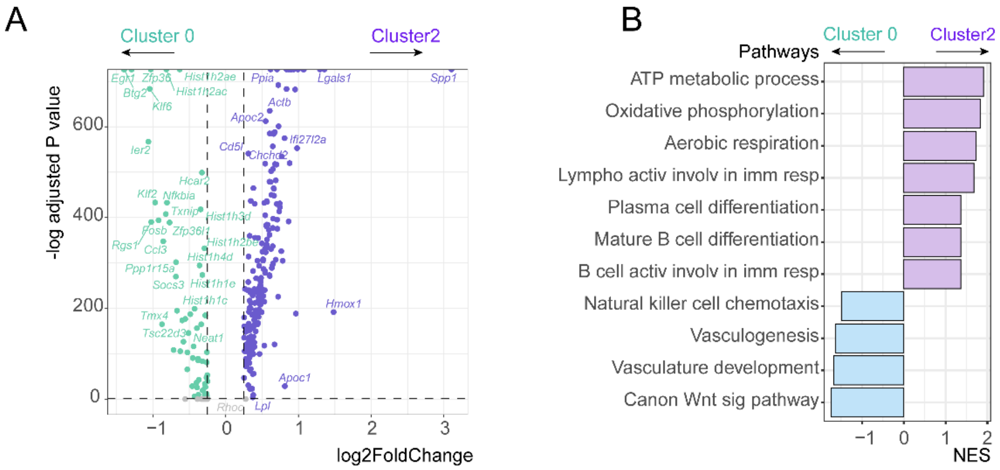

Supplementary Figure 21. Transcriptional profile comparison between microglia subcluster 2 and microglia subcluster 0. A. Differentially expressed genes between microglia subcluster 2 and microglia subcluster 0. B. GSEA analysis illustrating the significant pathways between microglia subcluster 2 and microglia subcluster 0. P value < 0.05 was considered statistically significant.

Reagent or resource	Source	Catalog number
Antibodies		
CD11b-FITC (1:100)	Biolegend	Cat#101206
CD45-APC (1:100)	Biolegend	Cat#147708
Rabbit-anti-Mouse-Iba-1 (1:500)	WAKO	Cat #019-19741
Goat-anti-Mouse-Iba-1 (1:500)	Novus Biologicals	Cat #NB100-1028
Rat-anti-Mouse-CD68 (1:500)	Bio-Rad	Cat #1957
FITC-Rat-anti-Mouse-IgM	eBioscience	Cat#11-5790-81
Rat-anti-Mouse-IgD	Biolegend	Cat#405702
Rat-anti-Mouse-CD138	Biolegend	Cat#142502
Alexa Fluor® 488 anti-mouse/human GL7 Antigen Antibody	Biolegend	Cat#144611
Rat-anti-Mouse-B220	Biolegend	Cat#103202
Rabbit-anti-Mouse-MBP	Abcam	Cat#40390
Rabbit-anti-Mouse-CD31	Abcam	Cat#ab222783
Rabbit-anti-Mouse-MAP2	Proteintech	Cat#17490-1-AP
Rabbit-anti-Mouse-OLIG2	R&D	Cat#AF2418
Rabbit-anti-Mouse-LGALS3	Biolegend	Cat#125401
Rat-anti-Mouse-CD4	CST Technology	Cat#96127
Rabbit-anti-Mouse-CD8	CST Technology	Cat#35467
Rabbit-anti-Mouse-FOXP3	CST Technology	Cat#12653
Rabbit-anti-Mouse-IFIT1	Proteintech	Cat#23247-1-AP
Rabbit-anti-Mouse-MIF	Abcam	Cat#ab187064
Rabbit-anti-Mouse-IL-6	Abcam	Cat#ab290735
Rabbit-anti-Mouse-IL-10	Abcam	Cat#ab313401
Rabbit-anti-Mouse-Ki67 (1:100)	Invitrogen	Cat #14-5698-37
CD11b microbeads (1:10)	Miltenyi biotec	Cat#130-093-636
PerCP-Cy5.5-Anti-mouse CD45	Biolegend	Cat#103131

BV421-Rat-anti-Mouse IgM	BD Pharmingen	Cat#562595
BV786-Rat-anti-Mouse IgD	BD Pharmingen	Cat#563618
Fixable Viability Stain 700	BD Pharmingen	Cat#564997
Fixable Viability Stain 780	BD Pharmingen	Cat#565388
PE-Rat-anti-Mouse CD138	BD Pharmingen	Cat#561070
APC-Rat-anti-Mouse CD19	Biolegend	Cat#115512
BV650 Rat-anti-Mouse CD19	BD Pharmingen	Cat#563235
FITC-Rat-anti-Mouse B220	CST Technology	Cat#34399
BV421-Rat-anti-Mouse CD1d	Biolegend	Cat#123527
PE-Rat-anti-Mouse CD5	Biolegend	Cat#100608
APC-Rat-anti-mouse Ki-67	Biolegend	Cat#652405
Purified (azide-free) F(ab')2 Goat anti-mouse lqM (u chain)	Biolegend	Cat#157102
BV510 Mouse-anti-Human CD19	BDBiosciences	Cat#562947
InvivoMab anti-mouse CD40	BioXCell	Cat#BE0016-2
MAP2-polyclonal antibody	Proteintech	Cat#17490-1-AP
InVivoMab anti-mouse IFNAR-1	BioXCell	Cat#BE0241
PE Rat-anti-mouse CD45	Biolegend	Cat#147711
Purified Rat Anti-Mouse CD16/CD32 (Mouse BD Fc Block)(2.4G2) "	BD Pharmingen	Cat#553142
Chemicals, peptides, and recombinant proteins		
LFB staining kit	ServiceBio	Cat #G1030
PrimeScript™ RT Master Mix	TAKARA	Cat #RR036A

Hieff® qPCR SYBR Green Master Mix	Yeasen	Cat # 11201ES03
Seahorse glycolytic rate assay	Agilent	Cat #103344-100
Seahorse mitostress assay	Agilent	Cat #103015-100
Seahorse fatty acid oxidation assay	Agilent	Cat #103672-100
XFe testing plate	Agilent	Cat #102342-100
Seahorse XF DMEM testing medium	Agilent	Cat #103575-100
Seahorse XF Glucose solution	Agilent	Cat #103577-100
Seahorse XF Pyruvate solution	Agilent	Cat #103578-100
Seahorse XF glutamine solution	Agilent	Cat #103579-100
MACS Tissue storage solution	Miltenyi	Cat#130-100-008
Lipofectamine RNAiMAX	ThermoFisher	Cat#13778030
Resiquimod	MedChemExpress	HY-13740
RPMI 1640 Cell culture medium	Boster	PYG0126
CellMax Fetal Bovine Serum	CellMax	SA211.02
Tamoxifen	MedChemExpress	Cat #HY-13757A
Collagenase D	Roche	11088858001
Flowcytometry tube	BDFalcon	Cat#352235
Adult brain dissociation kit	Miltenyi Biotec	Cat #130-107-677
Rneasy micro kit	Qiagen	Cat #74004
Cell strainer	Falcon	Cat#352350
7-AAD	BD Bioscience	Cat #559925
MS column	Miltenyi Biotec	Cat # 130-042-201
LS column	Miltenyi Biotec	Cat # 130-042-401
BD Pharmingen™ Transcription Factor Buffer Set	BDbiosciences	Cat # 562574
B cell isolation kit	Miltenyi	Cat # 130-090-862
Oligonucleotides		

Primer, Table S10	This paper	Supplementary table 10
Experimental models: Organisms/strains		
WT C57BL/6 mice	Hunan SJA Laboratory Animal Co. Ltd, Hunan, China.	Hunan SJA Laboratory Animal Co. Ltd, Hunan, China.
Cd19-DTR-eGFP	Shanghai Model Organisms Center, Inc	Shanghai Model Organisms Center, Inc
Cx3cr1CreER mice(B6.129P2(Cg)-Cx3cr1tm2.1(cre/ERT2)Litt/WganJ)	The Jackson Laboratory	Stock No: 021160
Software and algorithms		
GraphPad Prism 9	GraphPad Software. Inc.	https://www.graphpad.com/
Imaris 9	Bitplane	https://imaris.oxinst.com/
R 4.04	R	https://www.r-project.org/
ImageJ (Fiji)	NIH	https://imagej.nih.gov/ij/
R package Seurat 4	Stuart et al, 2019 [1]	https://satijalab.org
R package clusterProfiler v4.05	Wu et al, 2021 [2]	
R package monocle v3	Trapnell et al, 2014 [3]	Monocle (cole-trapnell-lab.github.io)

Supplemental Table 10

1. Stuart, T., Butler, A., Hoffman, P., Hafemeister, C., Papalexi, E., Mauck, W.M., 3rd, Hao, Y., Stoeckius, M., Smibert, P., and Satija, R. (2019). Comprehensive Integration of Single-Cell Data. Cell *177*, 1888-1902 e1821. 10.1016/j.cell.2019.05.031.
2. Wu, T., Hu, E., Xu, S., Chen, M., Guo, P., Dai, Z., Feng, T., Zhou, L., Tang, W., Zhan, L., et al. (2021). clusterProfiler 4.0: A universal enrichment tool for interpreting omics data. Innovation (Camb) *2*, 100141. 10.1016/j.xinn.2021.100141.
3. Trapnell, C., Cacchiarelli, D., Grimsby, J., Pokharel, P., Li, S., Morse, M., Lennon, N.J., Livak, K.J., Mikkelsen, T.S., and Rinn, J.L. (2014). The dynamics and regulators of cell fate decisions are revealed by pseudotemporal ordering of single cells. Nat Biotechnol *32*, 381-386. 10.1038/nbt.2859.
4. Jakel, S., Agirre, E., Mendanha Falcao, A., van Bruggen, D., Lee, K.W., Knuesel, I., Malhotra, D., Ffrench-Constant, C., Williams, A., and Castelo-Branco, G. (2019). Altered human oligodendrocyte heterogeneity in multiple sclerosis. Nature *566*, 543-547. 10.1038/s41586-019-0903-2.
5. Absinta, M., Maric, D., Gharagozloo, M., Garton, T., Smith, M.D., Jin, J., Fitzgerald, K.C., Song, A., Liu, P., Lin, J.P., et al. (2021). A lymphocyte-microglia-astrocyte axis in chronic active multiple sclerosis. Nature *597*, 709-714. 10.1038/s41586-021-03892-7.
6. Schirmer, L., Velmeshev, D., Holmqvist, S., Kaufmann, M., Werneburg, S., Jung, D., Vistnes, S., Stockley, J.H., Young, A., Steindel, M., et al. (2019). Neuronal vulnerability and multilineage diversity in multiple sclerosis. Nature *573*, 75-82. 10.1038/s41586-019-1404-z.
